# IL-18 deficiency ameliorates the progression from AKI to CKD

**DOI:** 10.1038/s41419-022-05394-4

**Published:** 2022-11-15

**Authors:** Junjun Luan, Jingqi Fu, Congcong Jiao, Xiangnan Hao, Zixuan Feng, Lingzi Zhu, Yixiao Zhang, Guangyu Zhou, Hongyu Li, Wei Yang, Peter S. T. Yuen, Jeffrey B. Kopp, Jingbo Pi, Hua Zhou

**Affiliations:** 1 Department of Nephrology, Shengjing Hospital of China Medical University (CMU), Shenyang, China; 2 Program of Environmental Toxicology, School of Public Health, CMU, Shenyang, China; 3 Department of Urology, Shengjing Hospital of CMU, Shenyang, China; 4 Shensu Science & Technology Co., Ltd, Suzhou, China; 5 Renal Diagnostics and Therapeutics Unit, NIDDK/NIH, Bethesda, MD USA; 6 Kidney Disease Section, NIDDK/NIH, Bethesda, MD USA

**Keywords:** Acute kidney injury, Chronic kidney disease

## Abstract

Inflammation is an important factor in the progression from acute kidney injury (AKI) to chronic kidney disease (CKD). The role of interleukin (IL)-18 in this progression has not been examined. We aimed to clarify whether and how IL-18 limits this progression. In a folic acid induced renal injury mouse model, we studied the time course of kidney injury and renal IL-18 expression. In wild-type mice following injection, renal IL-18 expression increased. In parallel, we characterized other processes, including at day 2, renal tubular necroptosis assessed by receptor-interacting serine/threonine-protein kinase1 (RIPK1) and RIPK3; at day 14, transdifferentiation (assessed by transforming growth factor β1, vimentin and E-cadherin); and at day 30, fibrosis (assessed by collagen 1). In IL-18 knockout mice given folate, compared to wild-type mice, tubular damage and necroptosis, transdifferentiation, and renal fibrosis were attenuated. Importantly, IL-18 deletion decreased numbers of renal M1 macrophages and M1 macrophage cytokine levels at day 14, and reduced M2 macrophages numbers and macrophage cytokine expression at day 30. In HK-2 cells, IL-18 knockdown attenuated necroptosis, transdifferentiating and fibrosis.In patients with tubulointerstitial nephritis, IL-18 protein expression was increased on renal biopsies using immunohistochemistry. We conclude that genetic IL-18 deficiency ameliorates renal tubular damage, necroptosis, cell transdifferentiation, and fibrosis. The renoprotective role of IL-18 deletion in the progression from AKI to fibrosis may be mediated by reducing a switch in predominance from M1 to profibrotic M2 macrophages during the process of kidney repair.

## Introduction

Acute kidney injury (AKI) has become worldwide public problem that contributes to high morbidity and mortality in hospitalized patients [1]. AKI occurs in 10–15% all hospitalized patients [2] and in more than 50% in intensive care unit [3]. The prevalence or risk of AKI to CKD is high to 25% of all hospitalized AKI [4]. Better understanding of pathogenesis of AKI to CKD progression and developing effective therapeutic approaches will likely prevent or delay prognosis of AKI and reduce medical economic burden.

Inflammation and immune cells play important roles in AKI to CKD progression [5]. Interleukin (IL)-18, known as interferon-γ inducing factor [6], is well known as a key pro-inflammation cytokine and can be secreted by macrophages. IL-18 has reported to express in different types of renal cells such as tubular epithelial cells, intercalated cells, and tubulointerstitial cells as well as mesangial cells in lupus nephritis (LN) and participates in the pathogenesis of tubular and glomerular diseases [7–10]. These results suggest that IL-18 resulted kidney damage may depend on both renal resident cells and macrophages. The exact role of IL-18 in AKI and CKD remains unclear.

Recent report summarized that IL-18 has shown a key role in AKI and CKD. IL-18 promotes clinically relevant AKI induced by sepsis, nephrotoxins, and ischemia/reperfusion injury and glomerular diseases, as well as unilateral ureteric obstruction (UUO) [11]. IL-18 binding protein reduces renal tubular apoptosis in ischemic AKI and UUO as well as renal fibrosis two weeks after ischemia-reperfusion [12–14]. IL-18 receptor deletion ameliorates spontaneous kidney injury in MRL/lpr Mice [15]. However, the role of IL-18 in the different phases of AKI to CKD progression and its corresponding mechanisms remains unreported.

In this study, we used a mouse model of AKI to CKD progression induced by folic acid (FA) injection. The role and mechanism of IL-18 in the progression from AKI to CKD were studied in IL-18 knockout (KO) mice including necroptosis in the early phase of AKI, transdifferentiation in the middle phase of AKI to CKD progression, and fibrosis in the late phase of CKD. We also investigated the switching from M1 to M2 macrophages in this model.

## Materials and methods

### Mouse model

Animal studies were approved by Animal Care and Use Committee of China Medical University (15052111) following NIH animal guideline. All mice used for experiments were housed 4 per cage, and allowed free access to standard food and drinking water. Mice were maintained under a 12-hour light/dark cycle with fixed temperature at 24 °C and humidity (40–55%) in animal division of China Medical University. Female C57BL/6 wild‑type (WT) and IL‑18 KO C57BL/6 mice (40 weeks old, weight range, 27–32 g; *n* = 48) were purchased from Jackson Laboratory (stock no. C57BL/6:000664‑JAX; IL‑18 KO: 004130‑JAX).WT (*n* = 24) and IL-18 KO mice (*n* = 24) were injected peritoneally with 250 mg/kg FA (Sigma-Aldrich, MO, USA) in the vehicle of 0.3 mM NaHCO3 (0.2 mL/mouse) as described previously [16].

Peripheral blood was collected on day 0, day 2, day 14 and day 30 following FA administration. Urine was collected on day 0, day 7, day 14 and day 30. Mice were anesthetized by sodium pentobarbital peritoneal injection (90 mg/kg) for euthanasia and kidney samples were collected after perfusion with PBS to remove intrarenal blood on day 0, day 2, day 14 and day 30 (Fig. S1).

### HK-2 cells

The HK-2 cell line, a well-characterized human renal tubular epithelial cell line, was purchased from ATCC (Manassas, VA, USA) and was cultured in growth medium, DMEM/F-12 medium supplemented with 100 U/mL penicillin G, 100 ug/mL streptomycin, and 10% bovine calf serum at 37 °C in a humid atmosphere of 95% air and 5% CO2.

The cultured HK-2 cells were stimulated with 250 pg/mL recombinant human TGF-β (hTGF-β) (R&D SYSTEMS, MN, USA) for 12, 24, 48 h. The cells were collected to extract total RNA for IL-18 detection (the primers’ information in Table S1). Cells were transfected with 20 nm IL-18 siRNA (sense 5'-GAUUCUGACUGUAGAGUATT-3', antisense 5'-UAUCUCUAGUCAGAAUCTT-3') /scrambled (Syngentech, Beijing, China) combined with hTGF-β stimulated for 12, 24, 48 h respectively. The HK-2 cells were collected to isolate protein for RIPK1, RIPK3 and TGF- β1, vimentin, E-cadherin, and COL-1 examination (the antibodies’ information in Table S2).

### Human kidney tissues

Patients with biopsy-proven tubulointerstitial nephritis (TIN), including acute, subacute, and chronic cases (*n* = 3 in each group), were from the Nephrology Department of Shengjing Hospital between 2017 January and 2019 December. Diagnoses were made by two nephrology pathologist following TIN standard criteria [17]. Normal kidney tissue was obtained from patient with kidney tumor from Urology Department. Kidney tissues located at least 5 cm away from the tumors as normal control kidney [18]. All subjects provided written consent to participate in a research protocol approved by Institutional Review Boards (Table S3).

### Serology chemistry

Serum creatinine (Scr), blood urea nitrogen (BUN) was measured by an Architect c16000 device (Abbott, Chicago, IL, USA). Serum interferon-γ (IFN-γ) was measured by Interferon gamma Mouse enzyme-linked immunosorbent assay (ELISA) kit (Abcam, Cambridge, USA) according to the manufacturer’s instructions.

### Histology and Immunostaining

Kidney sections cut from human (2 µm) and mouse (3 µm) paraffin-embedded kidney tissue blocks were deparaffinized and rehydrated. Tissues were stained with periodic acid-Schiff (PAS), Masson-trichrome, TdT-mediated dUTP Nick-End Labeling (TUNEL) (Vazyme, Nanjing, China), immunohistochemistry (IHC), and immunofluorescent (IF) antibodies.

For PAS staining, quantitative scoring of tubular injury was conducted based on a semiquantitative method as our previous description [19]. In brief, 10 non-overlapping fields (×200 magnification) in both cortex and out stripe of outer medulla (OSOM) were semi-quantitated using the following five-point scoring system: zero for no injury, one for 1–25%, two for 26–50%, three for 51–75% and four for 76–100% of the injured area per field. Tubular injury was defined as loss of brush border, tubular epithelial cells necrosis, tubular atrophy, scarring surface of cortex, infiltrated inflammatory cells. Renal tubular injury score of each mouse was calculated as the average injury score of all fields.

Quantification of TUNEL-positive cells was performed on ten randomly selected fields (×200 magnification) in both cortex and OSOM for each kidney. The average number of TUNEL-positive cells per high power field was expressed as the extent of apoptosis [19].

For IHC, kidney sections were processed antigen retrieval with 10 mM sodium citrate (pH 6.2) and blocking nonspecific reaction with 5% goat serum (Zsbio, Beijing, China), followed by incubation with antibodies against vimentin, collagen 1(COL-1), F4/80, CD11c, CD206, CD68, and IL-18 (the antibodies information in Table S2) overnight at 4 °C. After washing, sections were incubated with biotin-conjugated goat IgG for 30 min at room temperature, and reacted with streptavidin-conjugated peroxidase for 30 min at room temperature followed by visualization with a DAB kit to (Zsbio, Beijing, China). Images were captured by microscopy (Nikon Corporation, Tokyo, Japan). To quantify protein expression, 10 randomly-selected fields per kidney were digitally imaged. The positive staining was measured by Image Pro Plus software and expressed as a percentage of positive area within the total captured area [19].

For IF, pretreated kidney sections were incubated with antibodies against transforming growth factor β1(TGF-β1) at 4 °C overnight, then incubated with Alexa-568 goat IgG (Thermo Scientific, Rockford, IL) at room temperature for 1 h. After washing, the slides were mounted with VECTASHIELD® Mounting Medium containing 4',6-diamidino-2- phenylindole (DAPI) (Vector Labs, Burlingame, CA). Images were captured by immunofluorescent microscopy (Nikon Corporation, Tokyo, Japan). For quantitative analysis, the integrated optical density (IOD) was analyzed with Image Pro Plus software and expressed as the percentage of positive area within the total captured area.

For double IF staining of IL-18 and aquaporin 1(AQP1), one cortex tubular cell-specific marker, pretreated kidney sections were incubated with antibodies against IL-18 at 4 °C overnight, then incubated with Alexa-488 goat IgG (Thermo Scientific, Rockford, IL) at room temperature for 1 h. After washing three times with PBS, the kidney slides were incubated with primary antibody against AQP1at 4 °C overnight. Following three washes with PBS, the slides were incubated with secondary antibody labeled with Alexa-568 for 1 h in the dark at room temperature, followed by PBS wash. After washing, the slides were mounted with DAPI. Images were captured by immunofluorescent microscopy (Nikon Corporation, Tokyo, Japan).

### Western blotting

Kidney total proteins were extracted using RIPA buffer with protease inhibitors, and the concentrations were determined by the bicinchonic acid assay. Equal amount of protein was separated by SDS-PAGE and transferred onto PVDF membranes (Milliproe Immobilon-P, German). After blocking with 5% milk, membranes were incubated with primary antibodies against receptor-interacting serine/threonine-protein kinase1(RIPK1), RIPK3, TGF-β1, vimentin, E-cadherin, COL-1, or F4/80 overnight at 4 °C. The blots were incubated with peroxidase-conjugated goat IgG for 60 min at room temperature. The antibody-antigen reactions were detected by High-sig ECL Western Blotting (WB) Substrate and visualized using the Tanon 5500 imaging system (Shanghai, China). The protein loading variation was normalized by antibodies against α-tubulin or GAPDH. The blot density was analyzed by NIH Image J software (Bethesda, MD). The protein level is expressed as the ratio of blot density from individual protein to a housekeeping antibody (antibodies information in Table S2).

### Quantitative PCR

Kidney total RNAs was extracted with Trizol reagent (Life Technologies, Carlsbad, CA). The concentration was measured with Nanodrop 2000 (ThermoFisher Scientific, Waltham, MA). RNA (50 ng) was subjected to reverse transcription using Prime Script RT Reagent Kit and followed by PCR with SYBR Premix Ex Taq (Takara, China) for IL-18, TGF-β1, and vimentin, COL-1, CD68, F4/80, CD11c, inducible nitric oxide synthase (iNOS), C-X-C motif chemokine ligand 10 (CXCL10), IL-4, CD206, C-C motif chemokine ligand 26 (CCL26). Actin/GAPDH was used as an endogenous control gene. Primers were designed using Primer Express (Applied Biosystems, Carlsbad, CA) and synthesized by Life Technologies (Shanghai, China) (primer information in Table S1). Real time fluorescence was detected with QuantStudio 6 Flex quantitative PCR system (Applied Biosystems, Carlsbad, CA). The mRNA levels were expressed as 2^− ΔΔCt^ (ΔCt: Gadph Ct - individual gene Ct).

### Statistical analysis

Statistical software SPSS 22.0 (SPSS, Chicago, IL, USA) and Graphpad Prism 8.0 (Graphpad, San Diego, CA, USA) were used for statistical analysis and graphing. Quantitative data were expressed as mean ± SD. Differences between groups were analyzed for statistical significance by one-way or two-way ANOVA. *p* < 0.05 was considered as statistically significant.

## Results

### IL-18 deletion attenuated kidney injury following FA injection

**Fig. 1:**
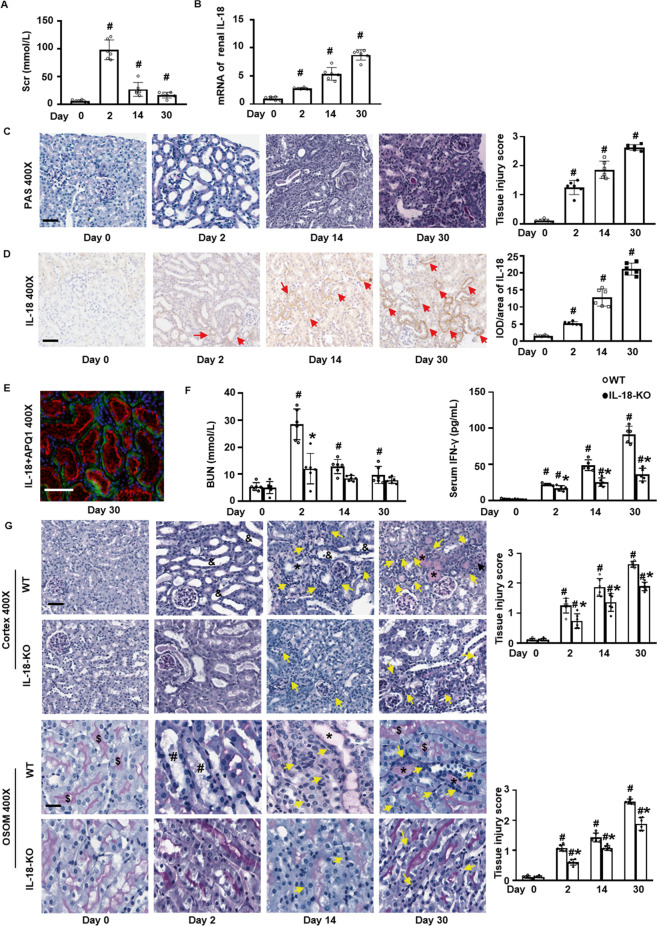
IL-18 deletion attenuates acute and chronic kidney injury in folic acid-induced mice. **A** The temporal changes of serum creatinine (Scr) and **B** renal mRNA of IL-18 expression following folic acid (FA) injection to wild type mice (WT). **C**, **D** kidney injuries on PAS staining and the semi-quantification of the injuries (**C**) and immunohistochemistry staining of IL-18 and its semi-quantification (**D**). **E** Double immunofluorescence staining of IL-18 (green) and AQP-1 (red) in the relative normal renal cortex tubular cells in WT mice at day 30 after FA injection. **F** Blood urea nitrogen (BUN) and Serum interferon-γ (IFN-γ) in IL-18 knockout mice (IL-18 KO) compared to WT mice. **G** PAS staining and its semi-quantification in renal cortex and outer medulla (OSOM). Red arrow indicates IL-18 positive staining. ^$^Normal brush border, ^&^loss of brush border and dilation of tubular lumen, *Protein cast, ^#^Detachment of tubular epithelial cells from tubular basement membrane and debris in tubular lumen. Yellow arrows indicate the infiltration of inflammatory cells. Magnification, 400 × bar = 50 um. Data represents Mean ± SD. (*n* = 6, ^#^*p* < 0.05, other groups vs. day 0; *p < 0.05, KO vs. WT).

In WT mice followed by FA injection, Scr and BUN peaked at day 2 and gradually returned to close to baseline level (Fig. 1A, F). The expression of renal IL-18 protein and mRNA progressively increased on qPCR and IHC (Fig. 1B and D). Further we found that co-localization of IL-18 and AQP1 in the relative normal renal cortex tubular cells in WT mice at day 30 after FA injection by double IF staining of IL-18 and AQP1 (Fig. 1E). PAS staining revealed acute kidney injury (AKI) at day 2, by loss of proximal tubule brush borders, detachment of tubular epithelial cells from tubular basement membrane, and dilation of tubular lumen. From day 14 to day 30, the inflammatory cells were infiltrated in the tubulointerstinum and were progressively increased along with the occurrence of patchy fibrosis and cortical surface scarring (Fig. 1C).

In IL-18 KO mice, AKI was attenuated, with lower by BUN at day 2 (Fig. 1F). Elevated serum IFN-γ can be induced by IL-18 [20], after FA injection, IL-18 was also lower compared to WT mice (Fig. 1F). The acute tubular damage at day 2 and increased infiltration of inflammatory cells in both renal cortex and outer stripe of outer medulla at day 14 and day 30 were also attenuated in IL-18 KO mice compared to WT mice (Fig. 1G). In addition, the elevated urine output and urine color changes on day 7 and day 14 was reverted to normal in IL-18 KO mice, compared to WT mice (Fig. S2).

### IL-18 deletion ameliorated necroptosis

**Fig. 2:**
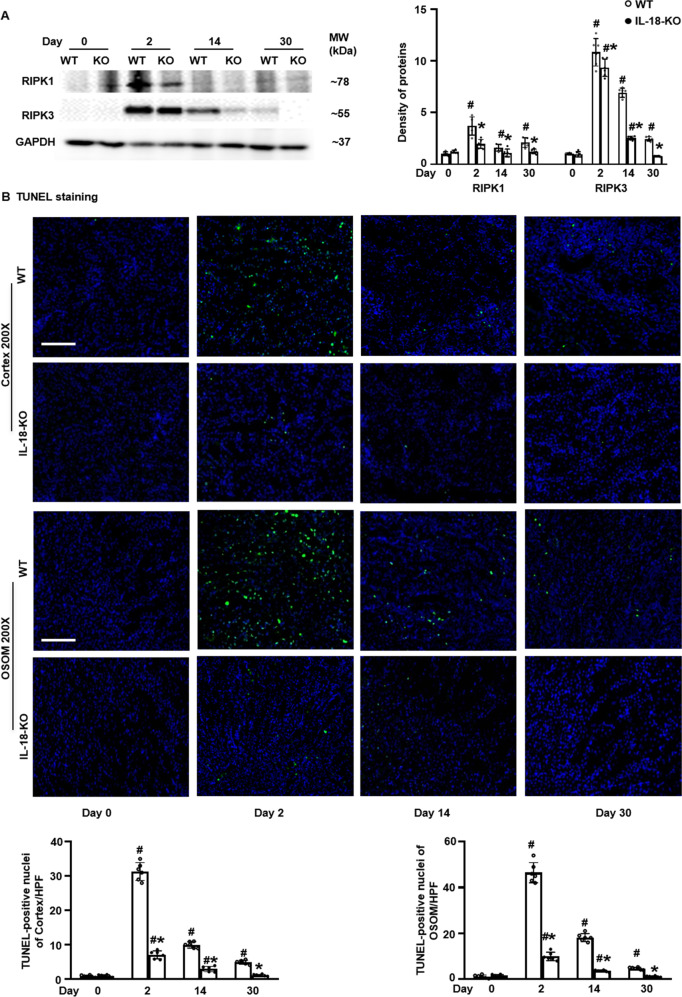
IL-18 deletion ameliorated renal necroptosis in folic acid-injected mice. **A** Renal protein expression of RIPK1 and RIPK3, two necroptosis biomarkers, peaked at day 2, analyzed by Western blotting and the density of the blots. **B** TUNEL staining and semi- quantitative analysis in renal cortex and outstrip of outer medulla (OSOM). Magnification, 200× bar = 100 um. Data represent Mean ± SD (*n* = 6, ^#^*p* < 0.05, other groups vs. day 0; *p < 0.05, KO vs. WT).

Necroptosis is an important form of cell death in inflammatory processes [21]. Renal expression of RIPK1 and RIPK3 peaked at day 2, then gradually decreased to day 14 and returned close to the baseline level at day 30 after FA injection in WT mice. Necroptosis was attenuated in IL-18-treated KO mice (Fig. 2A). TUNEL-positive cells, indicating programed cell death, showed similar as necroptosis cells in IL-18 deficiency mice compared to WT mice (Fig. 2B).

### IL-18 deletion suppressed tubular cell transdifferentiation during AKI repair

**Fig. 3:**
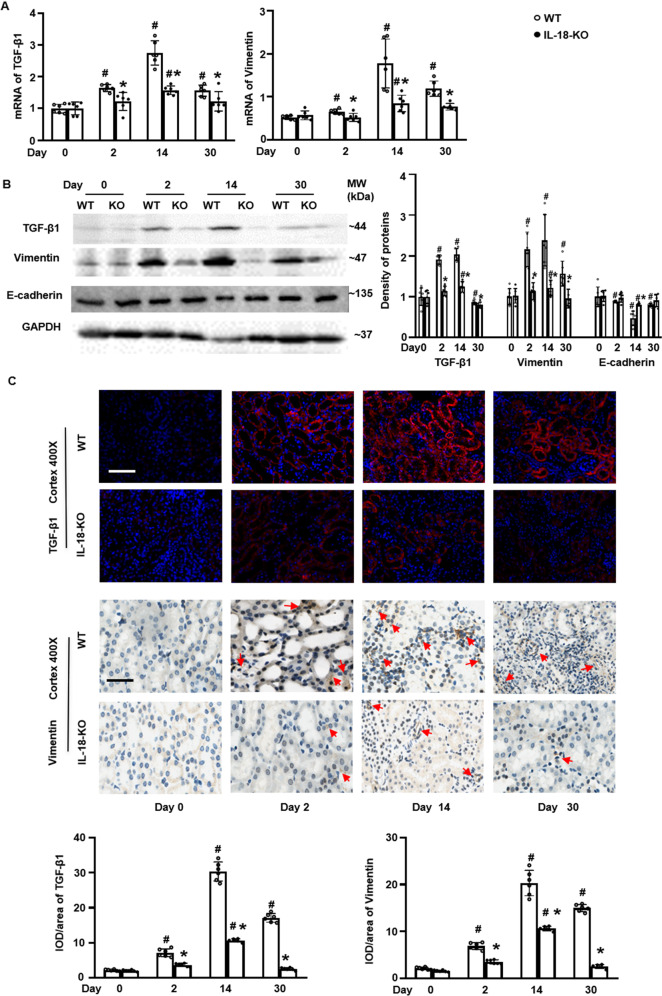
IL-18 deletion suppressed tubular cell transdifferentiation at day 14 after folic acid injection to mice. **A** Renal mRNA of TGF-β1, and Vimentin. **B** Renal proteins of TGF- β1, vimentin, and E-cadherin analyzed by Western blotting and the density of blots. **C** Immunofluorescent (IF) staining of TGF-β1 and immunohistochemistry (IHC) staining of vimentin (red arrows) and semi quantitative analysis in renal cortex. Magnification, 400×, bar = 50 um. Data represent Mean ± SD (*n* = 6, ^#^*p* < 0.05, other groups vs. day 0; *p < 0.05, KO vs. WT).

We examined expression of TGF- β1, vimentin, and E-cadherin, as well-known contributors of transdifferentiation during AKI repair [22–24]. The renal mRNAs and proteins of TGF- β1, and vimentin started to increase at day2, peaked at day 14, then decreased somewhat at day 30 but remained higher than the baseline after FA injection in WT mice. IL-18 deficiency attenuated these increases at each time points (Fig. 3A, B). This trend of TGF- β1 and vimentin was also seen on immunofluorescence staining of TGF-β1 and IHC staining of vimentin (Fig. 3C). At the same, we found that the renal E-cadherin protein expression started to decrease at day 2, reach to the nadir at day 14, then increased at day30 but still remained lower than the baseline level analyzed by Western Blotting (Fig. 3B).

### IL-18 deletion inhibited renal fibrosis in AKI transition to chronic injury

**Fig. 4:**
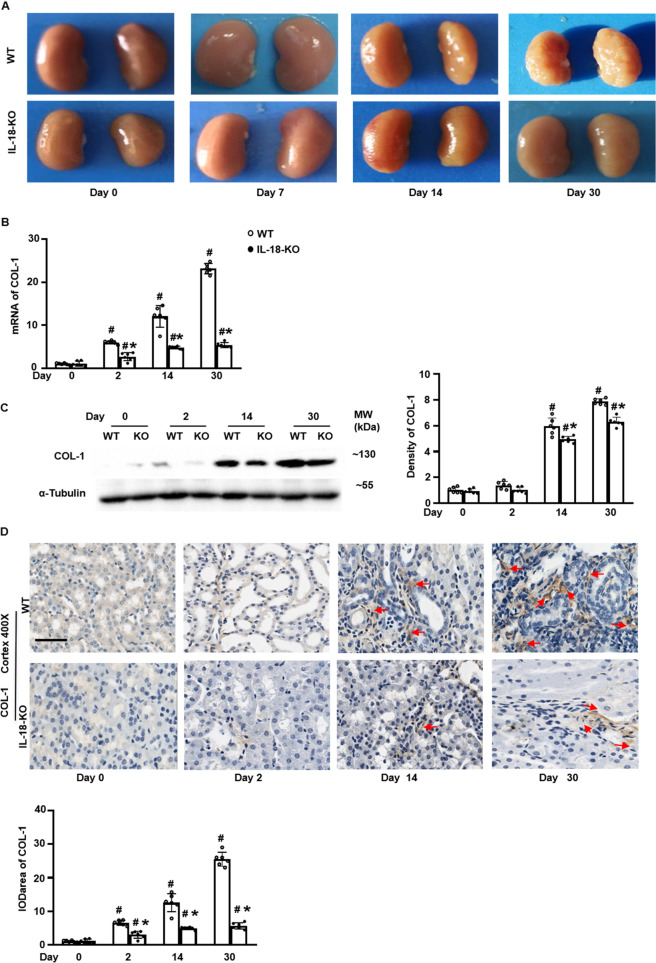
IL-18 deletion inhibited renal fibrosis induced by folic acid. **A** The kidney size started shrinking from day 14 and became much smaller at day 30 after folic acid injection. **B** Renal mRNA expression of COL-1. **C** Renal protein expression of COL-1 analyzed by Western blotting and its semi-quantification. **D** Immunohistochemistry (IHC) staining and semi-quantification of COL-1 (red arrows) in renal cortex. Magnification, 400×, bar = 50 um. Data represent Mean ± SD (*n* = 6, ^#^*p* < 0.05, other groups vs. day 0; *p < 0.05, KO vs. WT).

We found enlarged kidneys at day 2, and progressively shrinking kidneys from day 14 to day 30 after FA injection (Fig. 4A). This led us to examine fibrosis, assessed by COL-1 at day 30. COL-1 mRNA levels continued to increase from day 2 to day 30. COL-1 protein production progressively increased from day 14. IL-18 deletion suppressed production of COL-1 (Fig. 4B, C). The gradually increased renal COL-1 was also demonstrated by IHC staining (Fig. 4D). Renal fibrosis was also revealed by patchy fibrosis along medullar ray and scarring cortex surface on day 14 and day 30 on Masson staining. IL-18 deletion reverted the overexpression COL-1 and renal fibrosis (Fig. 4 and Fig. S3).

### IL-18 deletion decreased infiltration of total macrophages

**Fig. 5:**
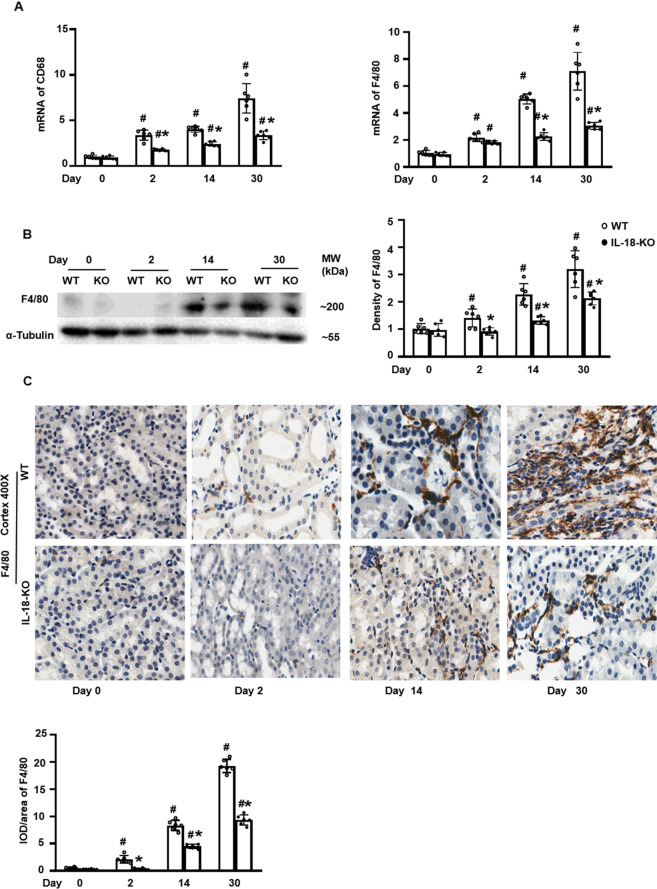
IL-18 deletion reduced the renal infiltration of total macrophages after folic acid injection. **A** mRNAs of total macrophages indicated by CD68 and F4/80 progressively increased after the folic acid injection. **B** Protein expression of F4/80 analyzed by Western blotting and its semi-quantification. **C** Immunohistochemistry (IHC) staining of F4/80 and semi-quantification in renal cortex. Magnification, 400×, bar = 50 um. Data represent Mean ± SD (*n* = 6, ^#^*p* < 0.05, other groups vs. day 0; *p < 0.05, KO vs. WT).

In order to investigate the mechanisms underlying the renal protective role provided by IL-18 deletion in the progression from AKI to CKD, we examined the renal infiltration of macrophages. We found mRNAs of macrophages, identified by CD68 and F4/80 expression, gradually increased from day 2 to day 30 after folic acid administration. The mRNA and protein levels of CD68 and F4/80 were reduced at each time points in IL-18 KO mice compared to WT mice (Fig. 5A–C).

### IL-18 deletion decreased infiltration of M1 and M2 macrophages in different time point

**Fig. 6:**
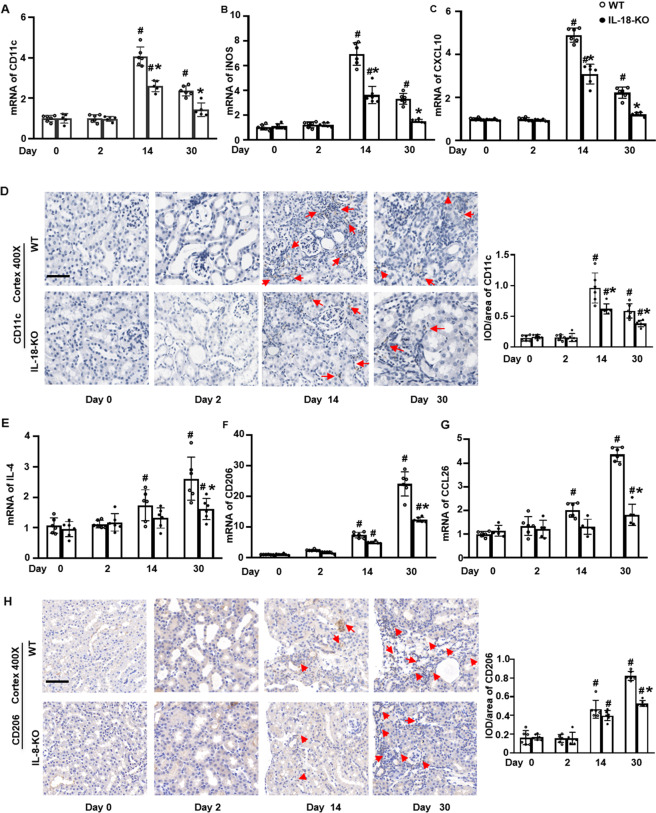
IL-18 deletion decreased renal infiltration of M1 and M2 macrophages triggered by folic acid injection. **A** CD11c, a M1 biomarker, peaked at day 14 and thenafter decreased at day 30. **B**, **C** M1 secretory factors such as iNOS, and CXCL10 showed similar change directions to CD11c. **D** Immunohistochemistry (IHC) staining of CD11c (red arrow) and its semi-quantification in renal cortex. **E** IL-4, an inducer of M2 from macrophage, **F** CD206, a M2 biomarker, responded same as IL-4. **G** CCL26, a M2 secreted cytokine were progressively increased and peaked at day 30 after folic acid injection analyzed by qPCR. **H** IHC staining of CD206 protein (red arrows) and its semi-quantification in renal cortex. Magnification, 400×, bar = 50 um. Data represent Mean ± SD (*n* = 6, ^#^*p* < 0.05, other groups vs. day 0; *p < 0.05, KO vs. WT).

We investigated the changes in macrophage subtype. M1 macrophages, indicated by CD11c, can express iNOS and can secrete CXCL10 [25]. Renal expression of CD11c mRNA started increasing at day 2, peaked at day 14, then decreased at day 30, on both mRNA and protein levels in WT mice after FA injection. The renal mRNAs of iNOS and CXCL10 showed similar changes and effects as CD11c in IL-18 KO mice compared to WT mice (Fig. 6A–D).

M2 macrophages can be stimulated by IL-4, represented by CD206, and can secret CCL26 [26]. The mRNA expression of these four molecules gradually increased from day 2 and peaked day 30. IL-18 deletion decreased this overexpression (Fig. 6E–G). The infiltration changes of M2 macrophages partially indicated by CD206 were also demonstrated by IHC staining (Fig. 6H).

### IL-18 knockdown attenuated necroptosis, transdifferentiating and fibrosis in vitro

**Fig. 7:**
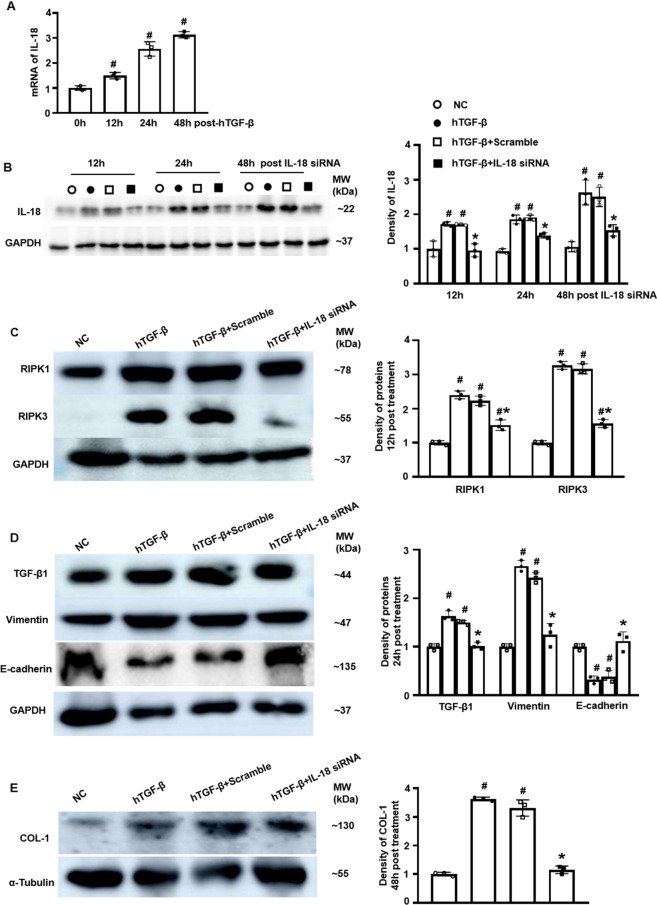
IL-18 knockdown attenuated necroptosis, transdifferentiation, and fibrosis in HK-2 cells. **A** The mRNA expression of IL-18 in HK-2 cells with hTGF-β stimulation by qPCR. **B** The protein expression of IL-18 HK-2 cells treated with hTGF-β and additional transfected with IL-18 siRNA and its scramble analyzed by Western blotting. **C** The protein expression of necroptosis indicated by RIPK1 and RIPK3 at 12 h after the transfection of IL-18 siRNA on Western blotting. **D** The expression of transdifferentiation marked by TGF- β1, vimentin, and E-cadherin at 24 h after the transfection of IL-18 siRNA on Western blotting. **E** The expression of fibrotic COL-1 at 48 h after the transfection of IL-18 siRNA on Western blotting (*n* = 3, #*p* < 0.05, other groups vs. NC; *p < 0.05, IL-18 siRNA vs. IL-18 scramble).

hTGF-β is a well-accepted as a stimulator to induce kidney tubular cells damage for investigating mechanisms in vitro study [27]. It is reported that cell death and fibrosis are increased in hTGF-β1-treated kidney tubular cells [28, 29]. We founded that IL-18 mRNA was gradually upregulated in HK-2 cells from 12 to 48 h after hTGF-β stimulation analyzed by qPCR (Fig. 7A). IL-18 siRNA alleviated the protein overexpression of IL-18 induced by hTGF-β on Western blotting (Fig. 7B). To investigate the effect of IL-18 downregulation on cell function, HK-2 cells were transfected with IL-18 siRNA /scrambled RNA with hTGF-β stimulation for 12, 24, 48 h respectively. The expression of necroptosis indicated by RIPK1 and RRPK3 were increased with hTGF-β stimulation, and IL-18 siRNA reverted the increased necroptosis from earlier point of 12 h after the siRNA transfection (Fig. 7C). The protein expression of transdifferentiation marked by TGF- β1and vimentin were also increased while E-cadherin was decreased in HK-2 cells with hTGF-β stimulation. The IL-18 siRNA reverted the expression of these proteins at 24 h after the transfection of siRNA (Fig. 7D). Similarly, the expression of COL-1, a fibrosis biomarker, was increased in HK2 cells with hTGF-β stimulation, and IL-18 siRNA reverted this overexpression at the late phasepoint of 48 h after siRNA transfection (Fig. 7E).

### IL-18 increased in renal biopsies from TIN patients

**Fig. 8:**
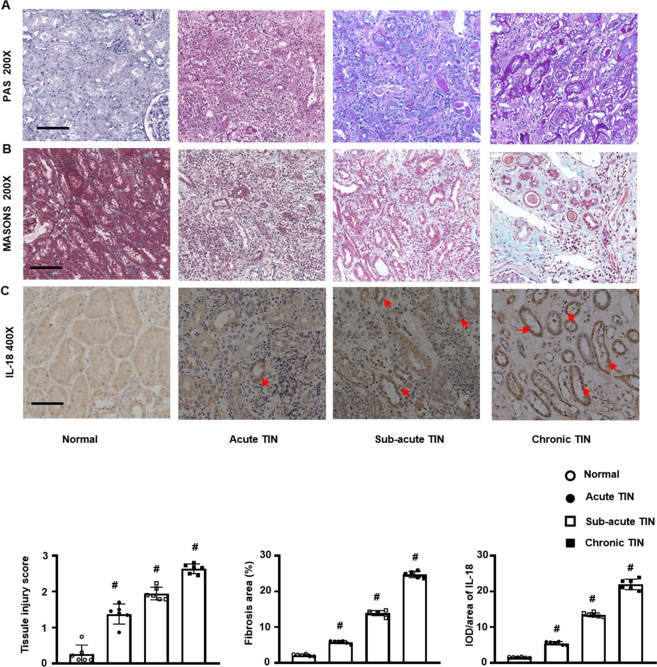
IL-18 increased in renal biopsies from patients with interstitial nephritis (TIN). **A** PAS staining. **B** Masson staining. **C** IHC staining of IL-18 (red arrow). Magnification, 200×, bar = 200 um.

Lastly, we studied IL-18 in clinically relevant disease applying TIN patients including acute, sub-acute, and chronic tubulointerstitial injury. Tubular damage, infiltrated interstitial inflammatory cells, and renal fibrosis showed a similar pattern of morphology to FA mouse model on PAS and Masson staining. IL-18 positive staining was gradually increased in acute, further augmented in sub-acute, and significant in chronic TIN (Fig. 8).

## Discussion

In present study, we mainly found the followings: (1) Renal IL-18 expression was elevated from day 2 to 30 in mice induced by FA injection and in renal biopsies from TIN patients. (2) IL-18 deletion attenuates FA-induced kidney injury from AKI to CKD progression. (3) IL-18 deficiency ameliorated renal necroptosis peaked at day 2, suppressed tubular cell transdifferentiation peaked at day 14, inhibited renal fibrosis peaked at day 30 after FA injection. (4) IL-18 deletion decreased infiltrated total macrophages including M1 macrophages peaked at day14 and M2 macrophages peaked at day 30.

IL-18 play a key role in AKI or CKD model animals [11]. IL-18 binding protein reduces tubular damage in ischemic AKI and renal fibrosis in late phase followed by ischemia/reperfusion and UUO [12–14]. IL-18 receptor deletion ameliorates tubular damage induced by sepsis, nephrotoxin, and UUO as well as glomerular injury in spontaneous LN and immunocomplex-induced glomerulonephritis [30–34]. Further IL-18 deletion shows renal protective role in mice of ischemic and nephrotic AKI as well as LN and glomerular nephritis [15, 35–37]. These results suggest that IL-18 deletion play a renal protective role. In this study, we found that IL-18 increased accompanied with kidney injury in mice model from AKI to CKD and TIN patients (Figs. 1 and 8). AKI is traditionally considered as reversible kidney injury. 25% of hospitalized AKI can progress to CKD and its corresponding mechanisms has been emphasized [4]. Various factors may participate the progression from AKI to CKD. including epidermal growth factor receptor, Nrf2 deficiency, hypoxia, ageing and, inflammatory cells [5, 19, 38–40]. However, the role of IL-18 in AKI to CKD progression remains unreported. FA-injected mice showed a typical progression from AKI to CKD [41, 42]. This is first study to investigate roles and its corresponding mechanism of IL-18 in FA-induced chronicity of AKI.

Necroptosis is a novel form of programmed cell death that has been identified as an important mechanism of inflammation-induced cell death [43]. Recently, two studies reported that necroptosis play important roles in cisplatin-induced AKI and polycystic disease [44, 45]. RIPK1 and RIPK3 promote necroptosis in intestinal epithelial cells [21]. There was no study about the role of IL-18 in necroptosis. In present study, RIPK1 and RIPK3 peaked at day 2 in WT mice after FA injection. IL-18 deficiency attenuated the increments of these necroptosis-related indices (Figs. 2 and 7). It suggested that early renal protective role of IL-18 deletion might be mediated by reducing necroptosis in early tubular damages induced by FA. Besides, it has been reported that ferroptosis mediated first wave of cell death in folic acid-AKI, and this promotes a second wave of cell death by necroptosis [46, 47]. The reduction of RIPK1 and RIPK3 levels may be because IL-18 prevents ferroptosis activation that needs further study.

Following AKI, damaged tubular cells may undergo transdifferentiation and may promote tissue repair or progressive renal fibrosis [40]. TGF-β1 and vimentin are key regulators in cell transdifferentiation [22, 23]. We found that renal levels of TGF-β1 and vimentin was increased and peaked at day 14 after folic acid injection. However, E-cadherin was decreased and reached a nadir at day 14 and recovered afterward after folic acid injection. This was similar to the founding of Yuan et al. in FA nephropathy [48]. These factors were considered as the main phase of transdifferentiation. IL-18 deficiency reversed the changes of thses transdifferentiation proteins at day 14 after FA-induced AKI (Fig. 3). These results suggested that transdifferentiation may trigger later fibrosis formation of AKI and that IL-18 deletion might reduce transdifferentiation, to further slowdown progression from AKI to CKD.

Tubulointerstitial fibrosis is a common feature of progressive CKD [49]. Inhibition of IL-18 reduces renal fibrosis after ischemia/reperfusion [13]. We also found that the expression of COL-1, a renal fibrosis biomarker, increased gradually and peaked at day 30 after FA injection. Overexpression of COL-1 was suppressed by IL-18 deletion (Fig. 4). This suggested that IL-18 deficiency attenuated renal fibrosis. Macrophages are present in renal fibrosis [50] and IL-18 is produced by macrophages [51]. However, it remains unclear how IL-18 affects macrophages in the progression from AKI to CKD.

We found that tissue macrophage number increased progressively as AKI progressed to CKD after FA injection. The numbers infiltrated of infiltrating macrophages decreased in IL-18 deficient mice (Fig. 5). Recent studies demonstrate that macrophages could manifest different phenotypes, including M1 macrophages, considered as pro-inflammatory cells promoting renal injury and M2 macrophages, regarded as anti-inflammatory cells inhibiting the response to tubular injury. The CD206^+^ subset of M2 macrophages is strongly associated with renal fibrosis [50].

In the present study, renal M1 macrophage numbers peaked at day 14 and then decreased at day 30, while M2 macrophages progressively increased after day 2 and peaked at day 30 after FA injection. IL-18 deletion reduced both M1 and M2 macrophages in kidneys preventing fibrosis starting renal fibrosis occurrence (Fig. 6). The inhibition of renal fibrosis by IL-18 deletion might be mediated via its suppression to the infiltration of infiltrated macrophages with different space-time special effect and phenotype specific features.

The association of the increase numbers M1 macrophages and increased expression of TGF-β1 and vimentin in the tubular transdifferentiation phase with IL-18 overexpression in WT mice will require further investigation. Further understanding the role of M1 macrophages in transdifferentiation might help to stop or slow the progression from AKI to CKD.

In conclusion, IL-18 deficiency ameliorated early phase necroptosis, middle phase transdifferentiation, and late phase fibrosis. The renalprotective role of IL-18 deficiency in the progression from AKI to CKD may be mediated by M1-M2 switching of macrophages.

## Supplementary information

Supplemental data

Full and uncropped western blots

aj-checklist

## Data Availability

The datasets used and/or analyzed during the current study are available from the corresponding author on reasonable request.
