# Supplementary material for: IL-18 deficiency ameliorates the progression from AKI to CKD
Source: Cell Death Dis. 2022 Nov 15;13(11):957. doi: 10.1038/s41419-022-05394-4 (PMC9666542; doi:10.1038/s41419-022-05394-4)
Supplement: Supplementary file 1 — Supplemental data [file 41419_2022_5394_MOESM1_ESM.pdf]

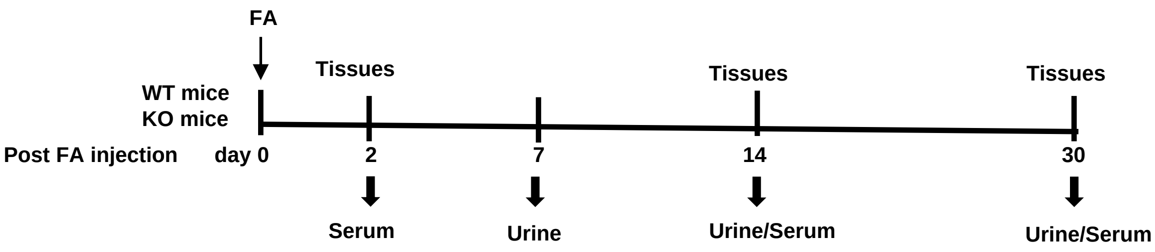

Figure S1. Experimental design. Mice were injected peritoneally by 250 mg/kg folic acid (FA). Peripheral blood samples were collected on day 0, day 2, day 14, and day 30 after FA administration. Urine samples were collected on day 0, day 7, day 14, and day 30. Kidney samples were collected after perfusion with PBS to remove intrarenal blood on day 0, day 2, day 14, and day 30.

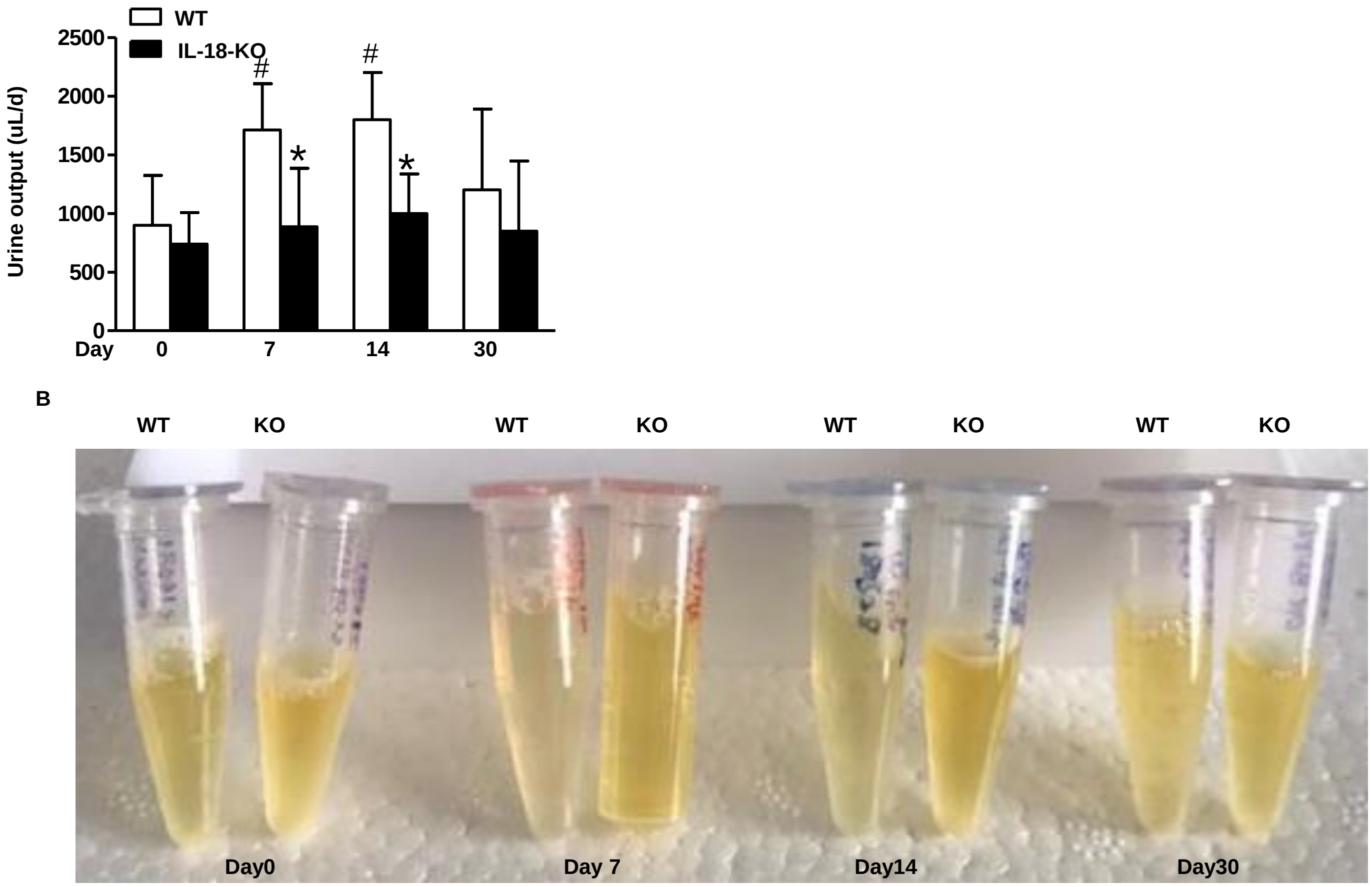

Figure S2. The effect of IL-18 deletion on urine output in folic acid-injected mice. (A-B) Urine output was increased on day 7 and 14 after folic acid (FA) injection wild type (WT) mice. In IL-18 knockout (KO) mice, the increment of urine output was attenuated. (B) The color of urine lightened on day 7 and day 14 and the color returned to the normal on day 30 after FA injection in WT mice. In IL-18 KO mice, urinary colors of urine samples from day 7 to day 30 after FA injection were close to normal on day 0. Data represent Mean ± SD (n=6, # $p<0.05$, other groups vs. day 0; * $p<0.05$, KO vs. WT)

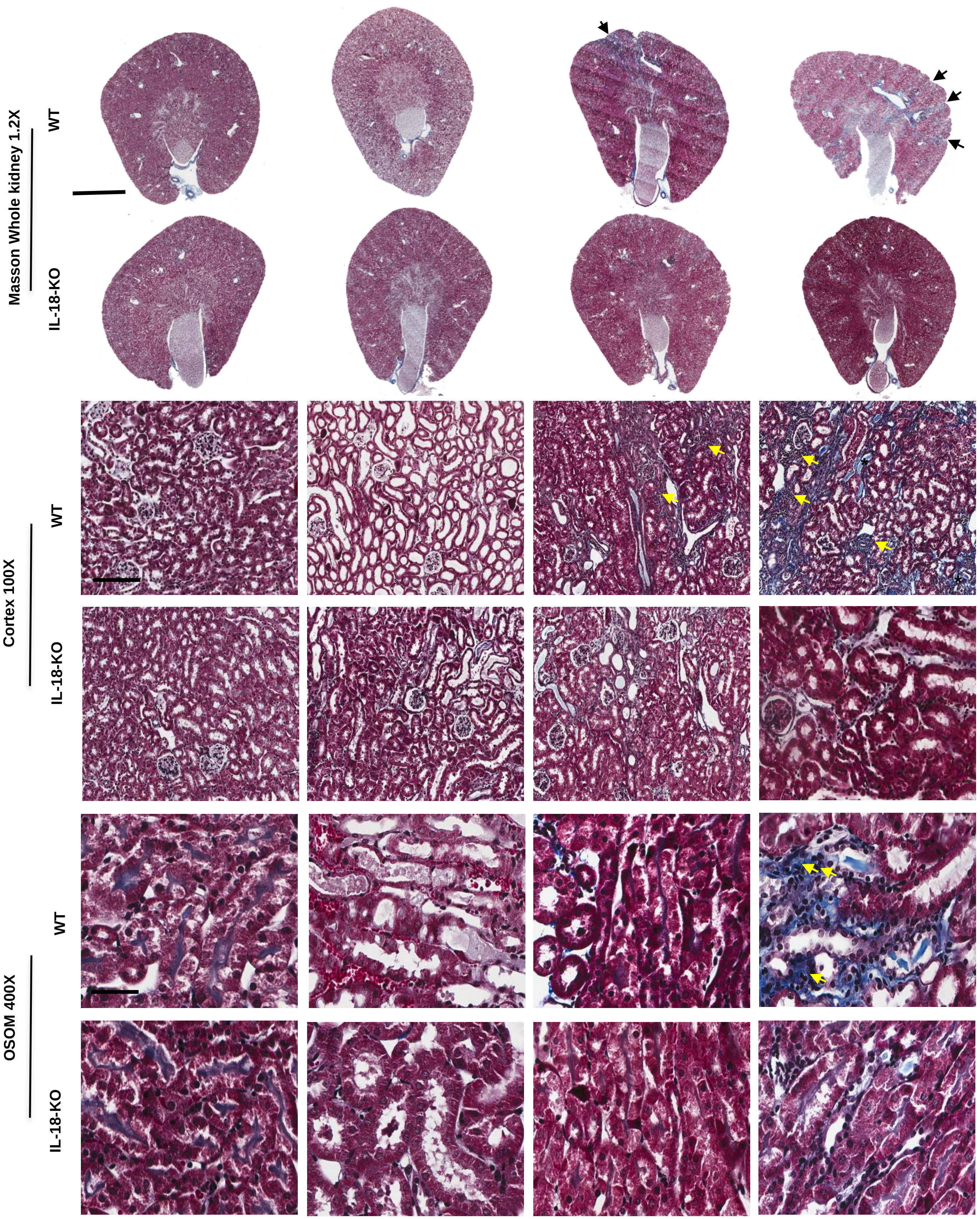

Figure S3. Masson staining of the kidneys in wild type mice and IL-18 knockout mice. The blue area indicates fibrosis.Blank arrows: fibrosis along patchy medullar ray and scarring cortex surface. Yellow arrows: the infiltration of inflammatory cells. (Magnification, 1.2X bar= 2000um;100X bar=200um; 400X bar = 50um)

Table S1. Sequence of primers used in qPCR

	Gene	Forward(5'-3')	Reverse(5'-3')
mouse	IL-18	GACTCTTGCGTCAACTTCAAGG	CAGGCTGTCTTTTGTCAACGA
mouse	TGF-β1	CTCCCGTGGCTTCTAGTGC	GCCTTAGTTTGGACAGGATCTG
mouse	Vimentin	CGTCCACACGCACCTACAG	GGGGGATGAGGAATAGAGGCT
mouse	COL-1	GACATGTTCAGCTTTGTGGACCTC	GGGACCCTTAGGCCATTGTGTA
mouse	CD68	TGTCTGATCTTGCTAGGACCG	GAGAGTAACGGCCTTTTTGTGA
mouse	F4/80	TGACTCACCTTGTGGTCCTAA	CTTCCCAGAATCCAGTCTTTCC
mouse	IL-4	GGTCTCAACCCCCAGCTAGT	GCCGATGATCTCTCTCAAGTGAT
mouse	CD206	CTCTGTTCAGCTATTGGACGC	CGGAATTTCTGGGATTCAGCTTC
mouse	CCL26	TTCTTCGATTTGGGTCTCCTTG	GTGCAGCTCTTGTCGGTGAA
mouse	iNOS	GTTCTCAGCCCAACAATACAAGA	GTGGACGGGTCGATGTCAC
mouse	CD11c	CTGGATAGCCTTTCTTCTGCTG	GCACACTGTGTCCGAACTCA
mouse	CXCL10	CCAAGTGCTGCCGTCATTTTC	GGCTCGCAGGGATGATTTCAA
mouse	Actin	TTCCTTCTTGGGTATGGAAT	GAGCAATGATCTTGATCTTC
human	IL-18	TCTTCATTGACCAAGGAAATCGG	TCCGGGGTGCATTATCTCTAC
human	GAPDH	GGAGCGAGATCCCTCCAAAAT	GGCTGTTGTCATACTTCTCATGG

Abbreviations: IL, interleukin ;TGF-β1, transforming growth factor β1; COL-1, collagen 1; CCL, C-C motif chemokine ligand; iNOS, in-ducible nitric oxide synthase ; CXCL, C-X-C motif chemokine ligand.

Table S2. Antibodies used in WB and IHC or IF

Protein	Company	Catalog	Host	Application	Dilution
RIPK1	Cell Signaling Technology	3493	Rabbit	WB	1:1000
RIPK3	Cell Signaling Technology	95702	Rabbit	WB	1:1000
TGF-β1	novusbio	NBP1-80289	Rabbit	WB	1:1000
				IF	1:200
VIMENTIN	Cell Signaling Technology	5741	Rabbit	WB	1:1000
				IHC	1:200
E-cadherin	Cell Signaling Technology	3195	Rabbit	WB	1:1000
COL-1	Thermo Fisher Scientific	PA1-26204	Rabbit	WB	1:1000
				IHC	1:200
F4/80	Santa Cruz Biotechnology	SC-25830	Rabbit	WB	1:1000
				IHC	1:200
CD11c	Cell Signaling Technology	97585	Rabbit	IHC	1:300
CD206	Cell Signaling Technology	91992	Rabbit	IHC	1:300
CD68	Abcam	Ab213363	Rabbit	IHC	1:200
IL-18	WanleiBio	WL01127	Rabbit	IHC/IF	1:200
				WB	1:1000
AQP1	Abcam	ab9566	Mouse	IF	1:300
α-TUBULIN	Santa Cruz Biotechnology	SC-5286	Mouse	WB	1:1000
GADPH	Santa Cruz Biotechnology	SC-25778	Rabbit	WB	1:1000
Goat anti Mouse IgG	Santa Cruz Biotechnology	SC-2039	Goat	WB	1:8000
Goat anti Rabbit IgG	Thermo Fisher Scientific	31460	Goat	WB	1:1000
Alexa Fluor® 568	Thermo Fisher Scientific	A11036	Goat	IF	1:200
Alexa Fluor® 488	Thermo Fisher Scientific	A11001	Goat	IF	1:200

Abbreviations: RIPK, receptor-interacting serine/threonine protein kinase; TGF-β1, transforming growth factor β1; COL-1 ,collagen 1; IL-18, interleukin 18; AQP1: Aquaporin 1; GADPH, glyceraldehyde-3-phosphate Dehydrogenase; WB, Western blotting ; IF, immunofluorescence; IHC, immunohistochemistry.

Table S3. The clinical characteristics of patients

No.	Age	Sex	eGFR(ml/min/1.73m^2)			Scr (umol/L)		
			At biopsy	3-month follow-up	6-month follow-up	At biopsy	3-month follow-up	6-month follow-up
1	47	F	117	-	-	56	-	-
2	58	M	127	-	-	62	-	-
3	60	M	125	-	-	62	-	-
4	51	F	8	10	12	500	420	360
5	28	F	12	15	10	430	355	500
6	64	M	12	9	8	330	415	460
7	52	F	9	20	18	450	230	255
8	28	M	24	28	12	290	255	515
9	59	M	3	9	7	1320	550	670
10	67	F	46	38	15	107	125	275
11	37	M	50	49	55	150	153	141
12	33	M	8	9	9	695	620	630

Abbreviations: eGFR, estimated glomerular filtration rate based on CKD-EPI formula. Scr, serum creatinine. No.1-3, normal controls. No.3-6, acute interstitial nephritis patients. No.7-9, sub-acute intersitial nephritis patients. No.10-12, chronic interstitial nephritis patients
