# Supplementary material for: IL-18 deficiency ameliorates the progression from AKI to CKD
Source: Cell Death Dis. 2022 Nov 15;13(11):957. doi: 10.1038/s41419-022-05394-4 (PMC9666542; doi:10.1038/s41419-022-05394-4)
Supplement: Supplementary file 2 — Full and uncropped western blots [file 41419_2022_5394_MOESM2_ESM.pdf]

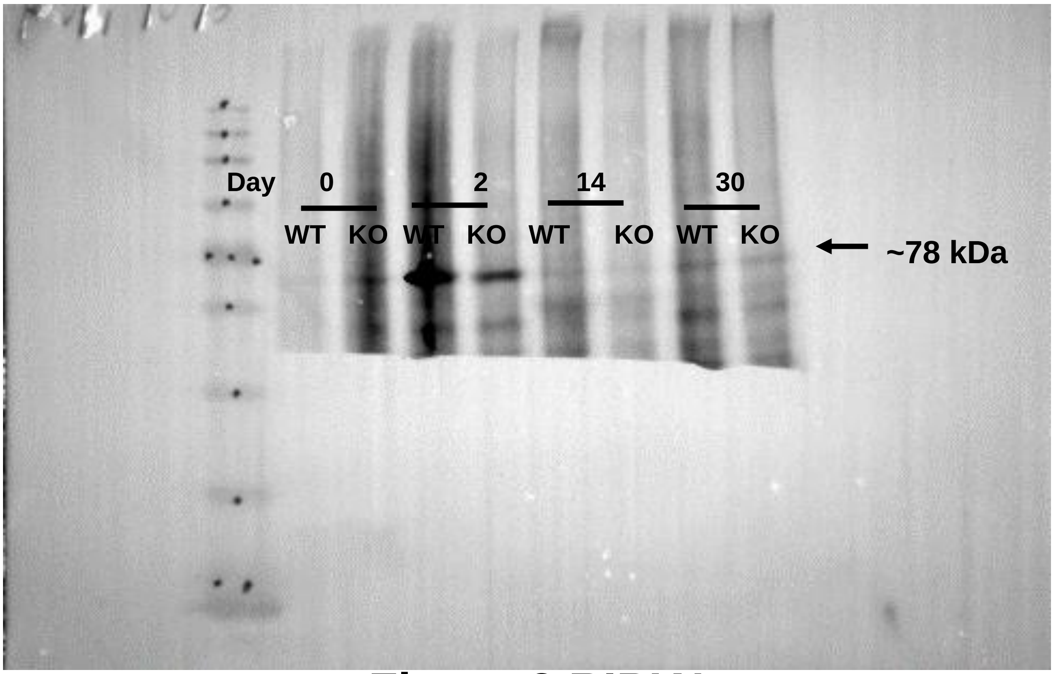

Figure 3 RIPK1

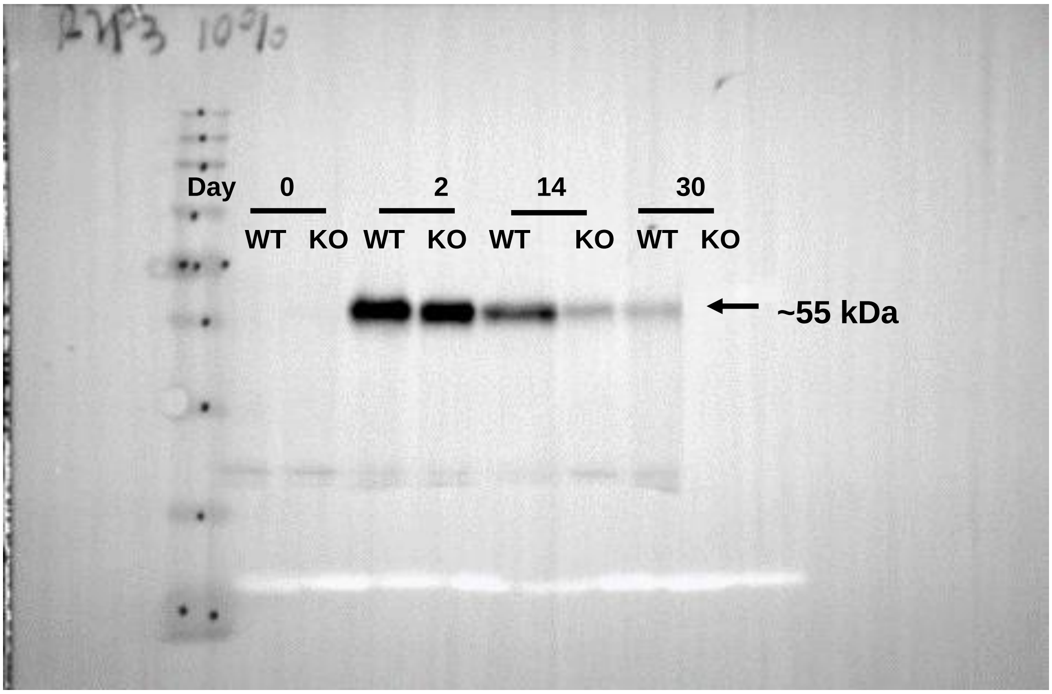

Figure 3 RIPK3

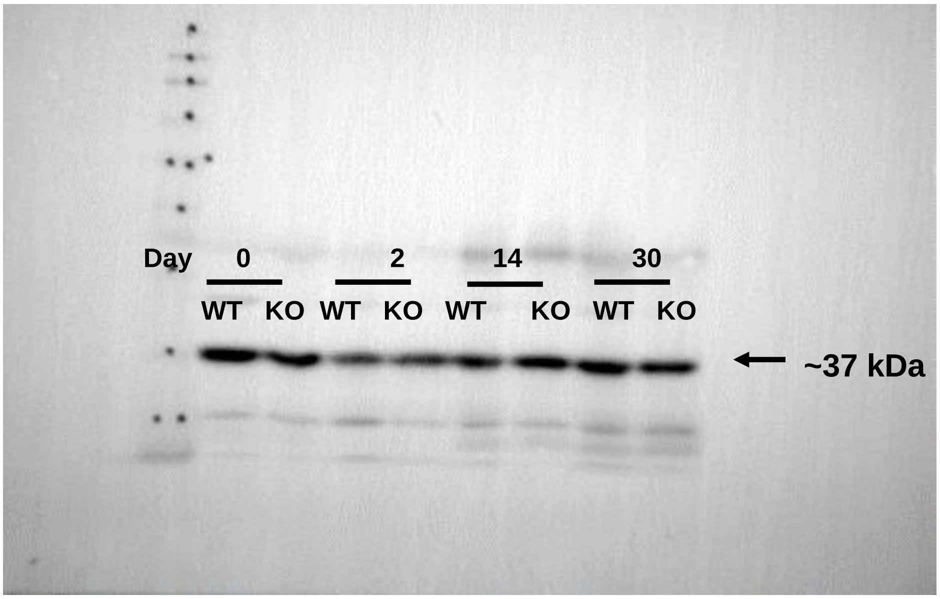

Figure 3 GAPDH

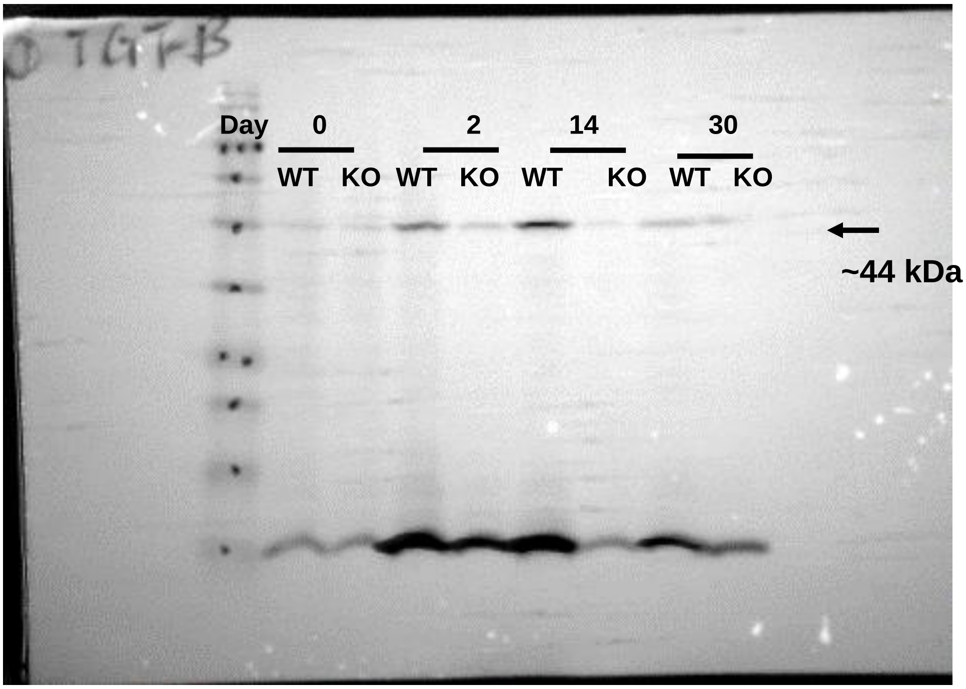

Figure 4 TGF-β1

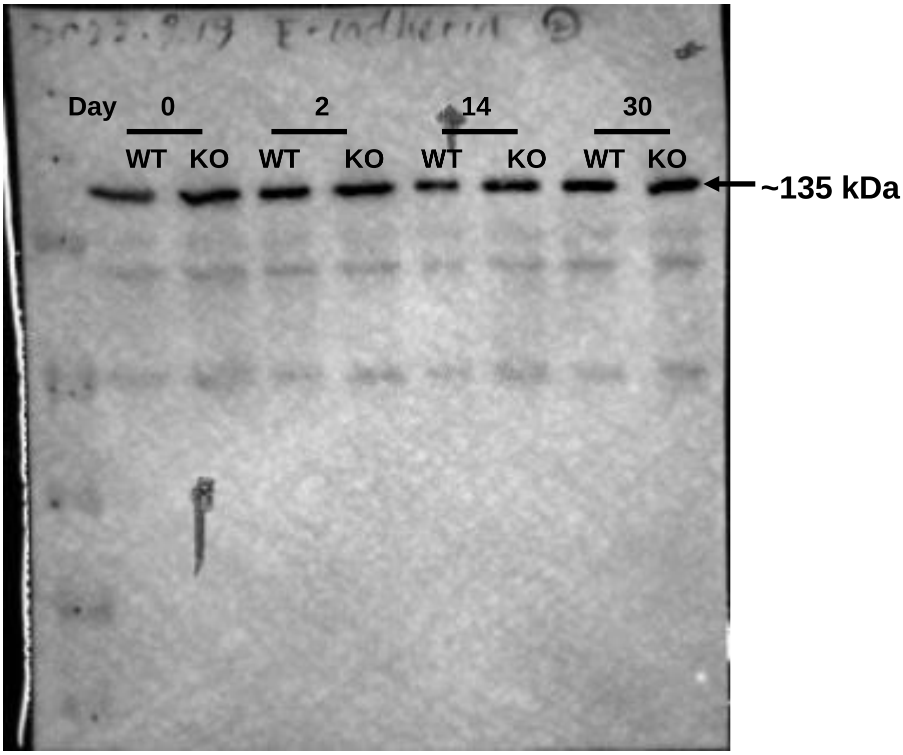

Figure 4 E-cadherin

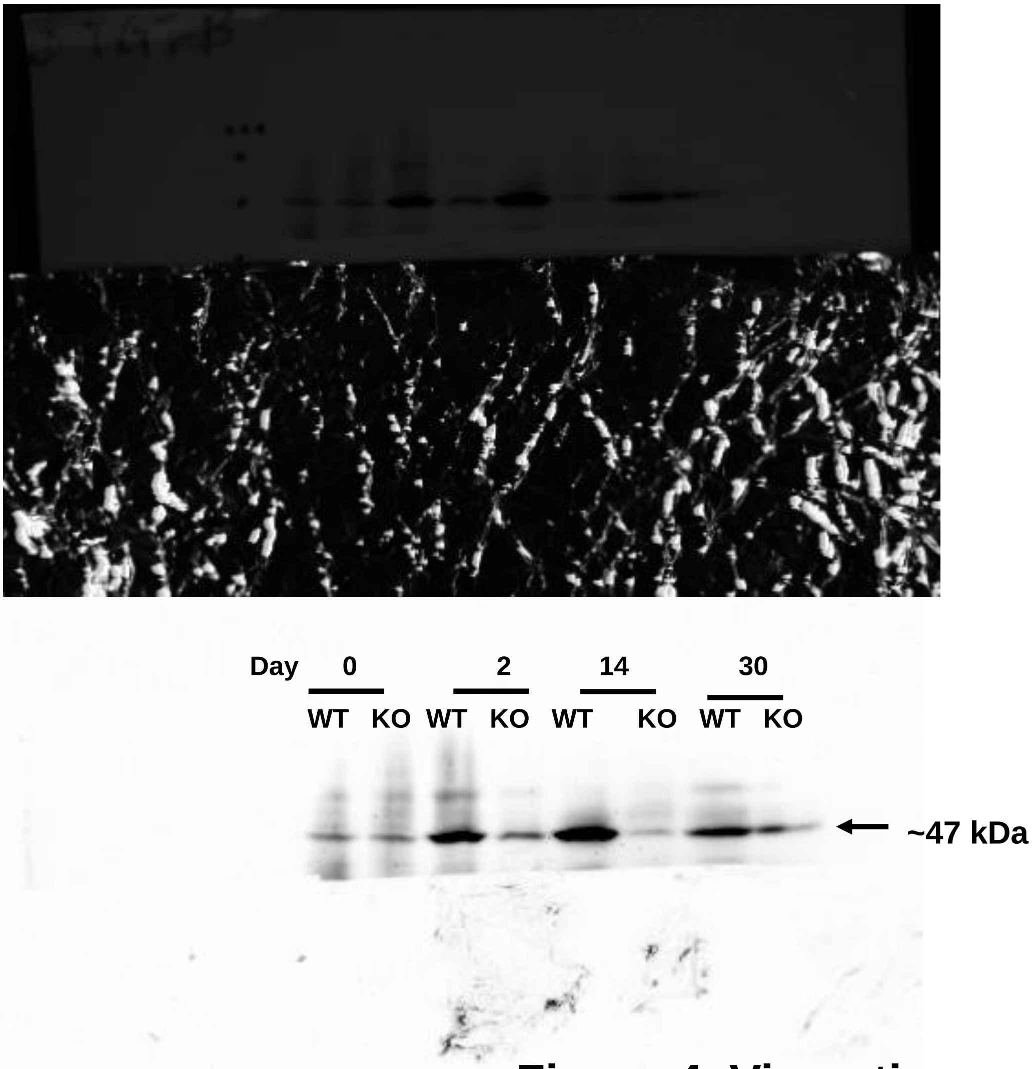

Figure 4 Vimentin

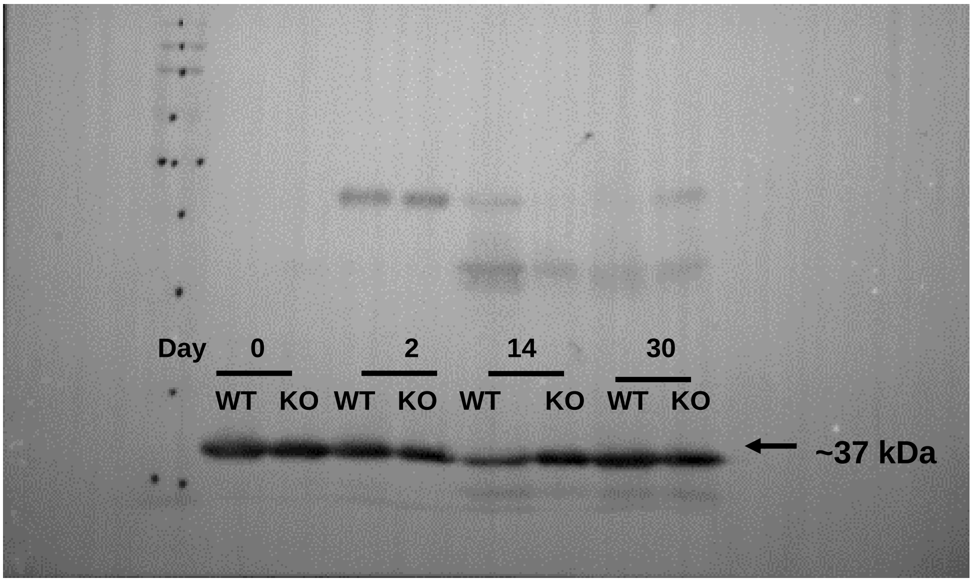

Figure 4 GAPDH

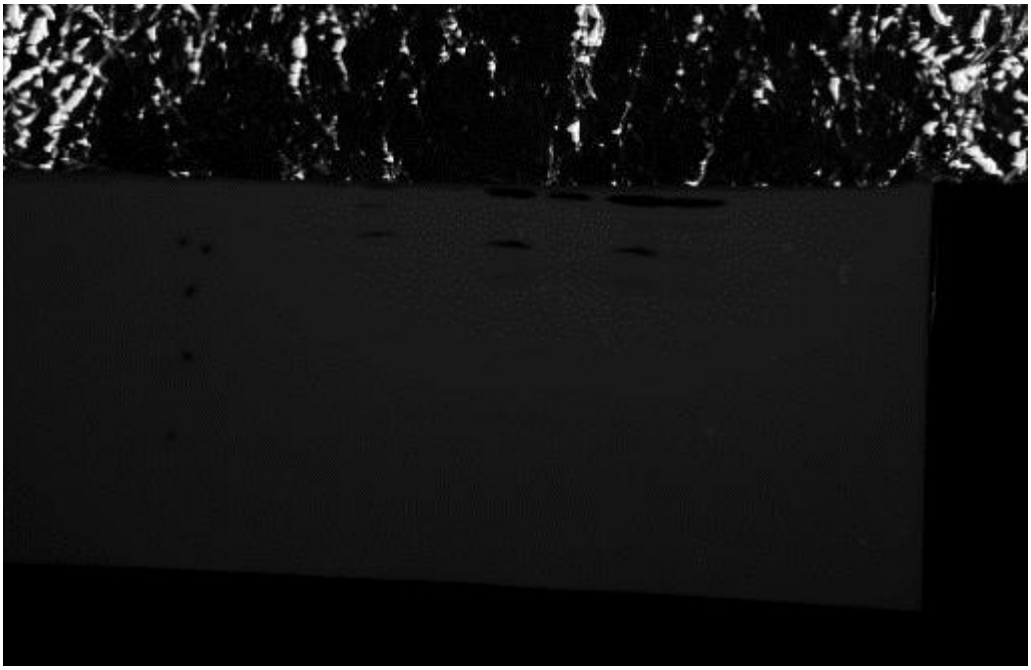

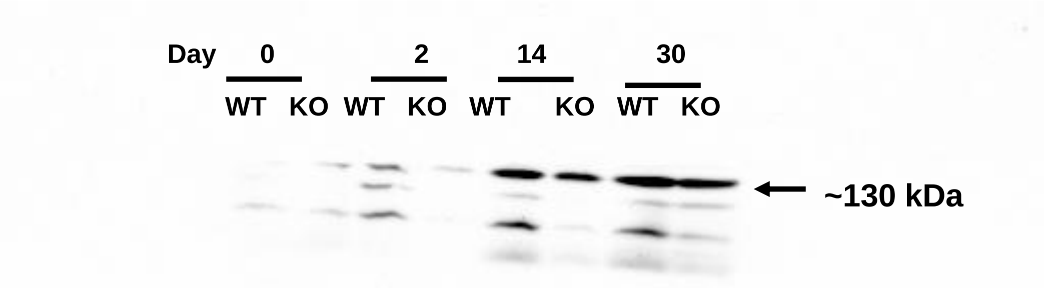

Figure 5 COL-1

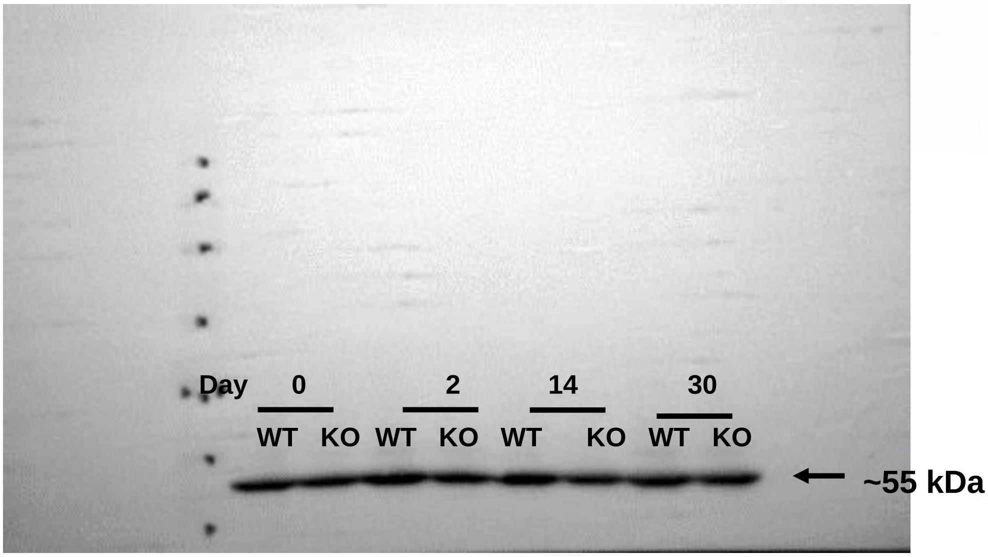

Figure 5 α-TUBULIN

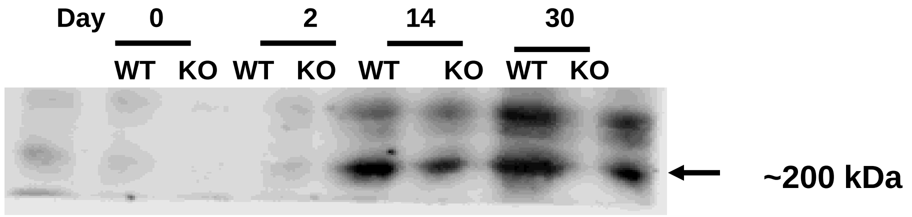

Figure 6 F4/80

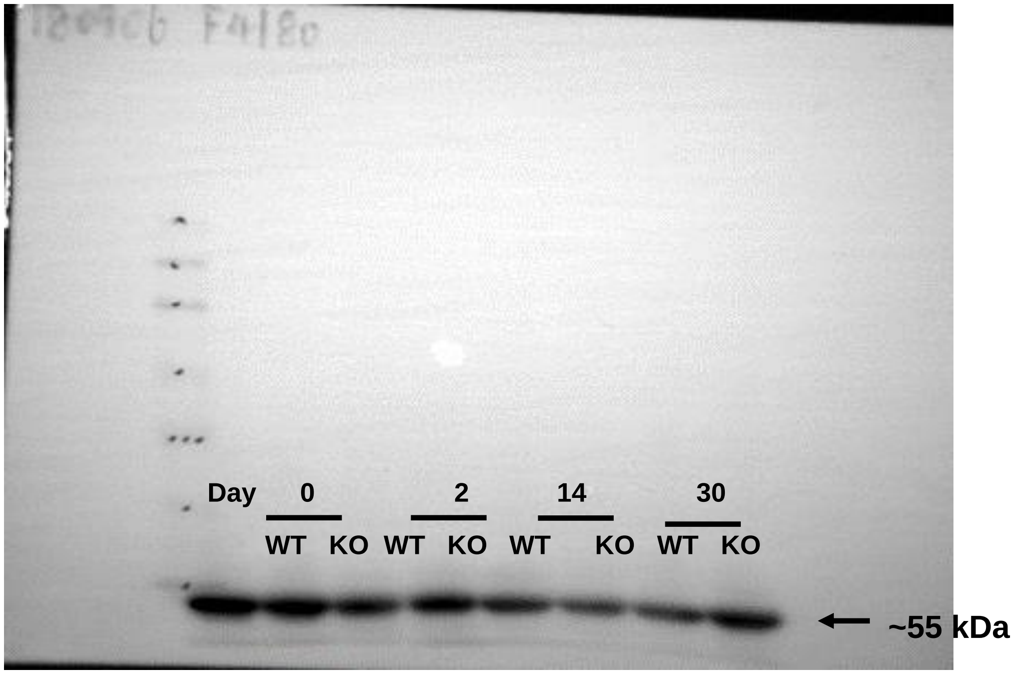

Figure 6 α-TUBULIN

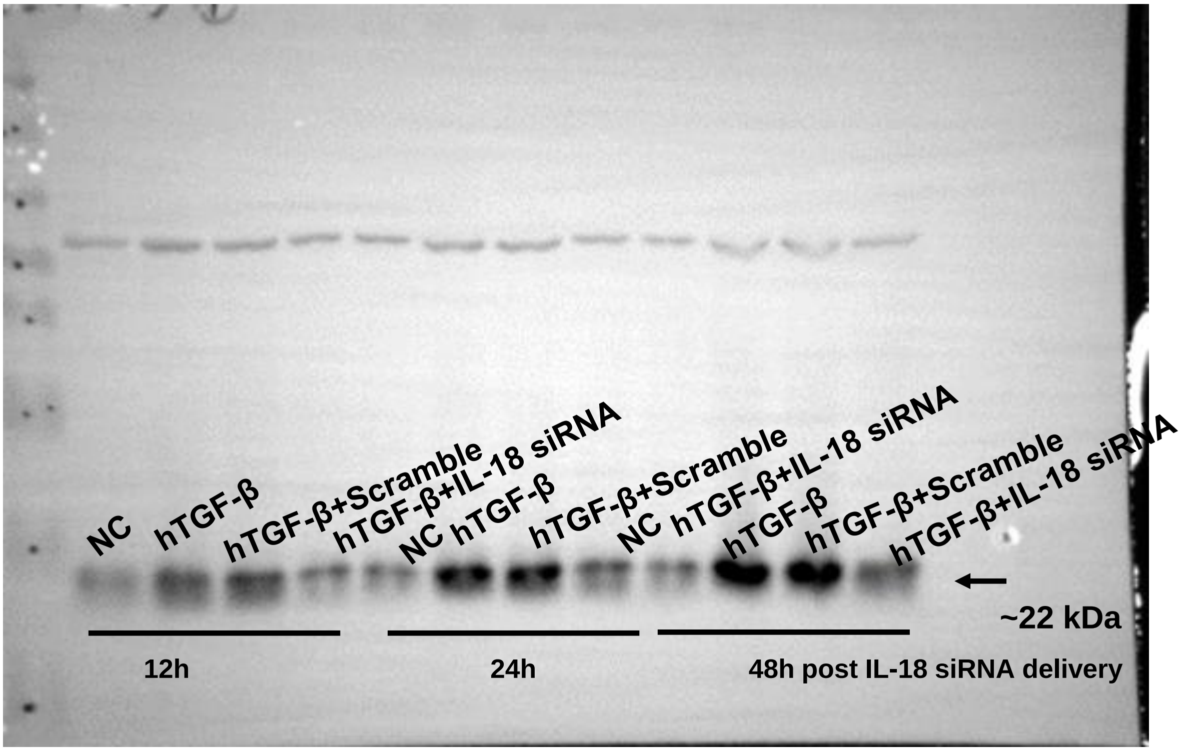

Figure 7 IL-18

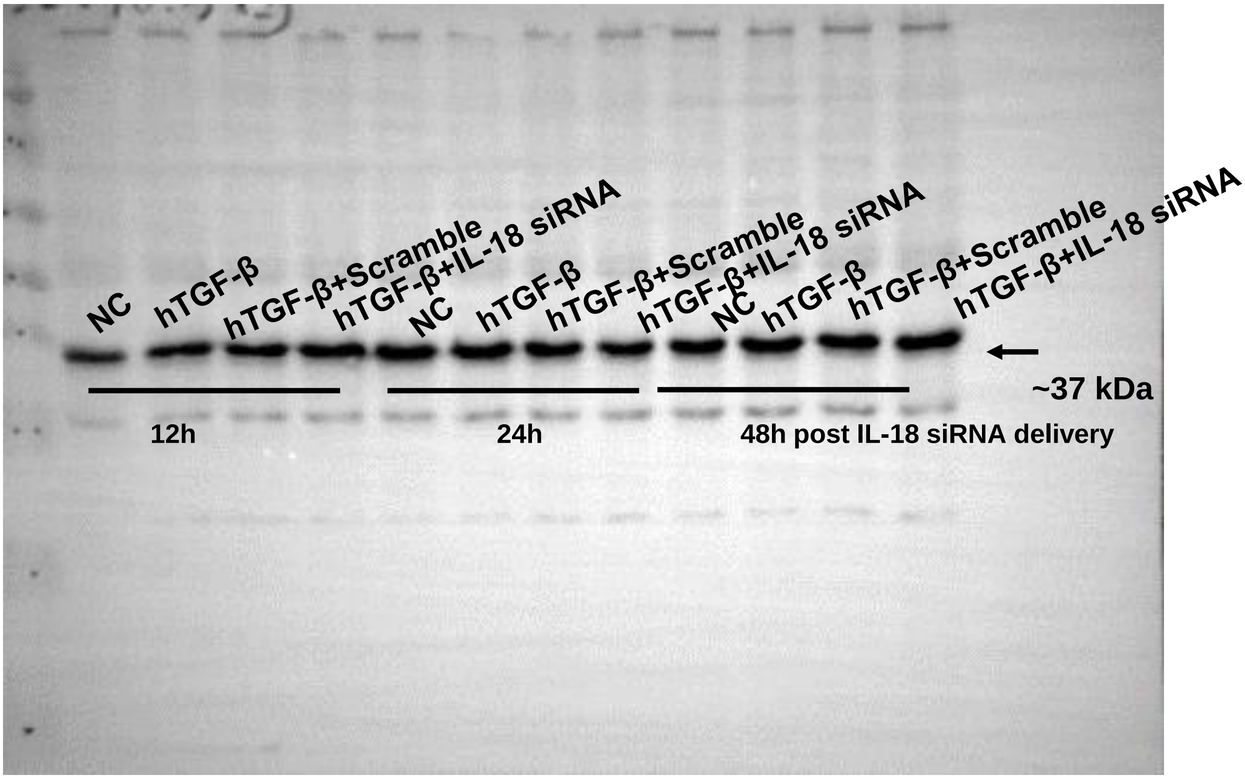

Figure 7 GAPDH

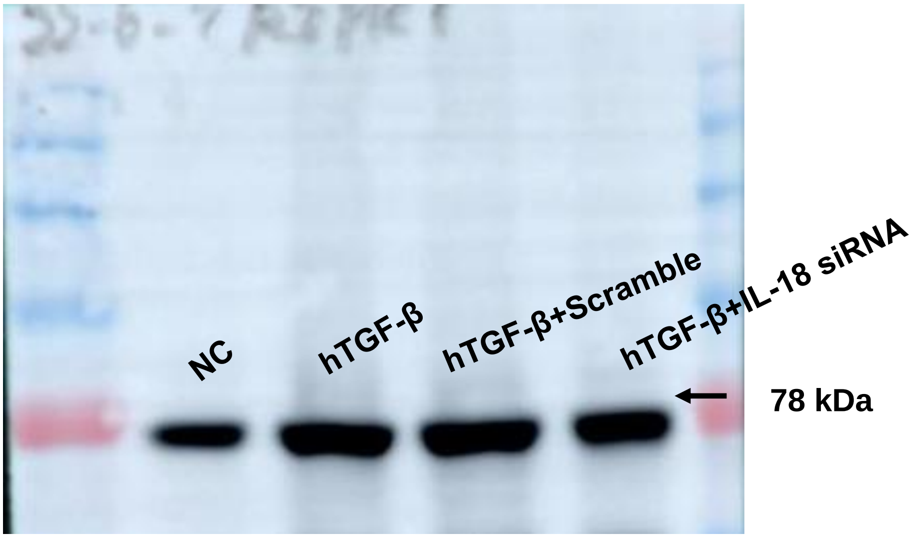

Figure 7 RIPK1

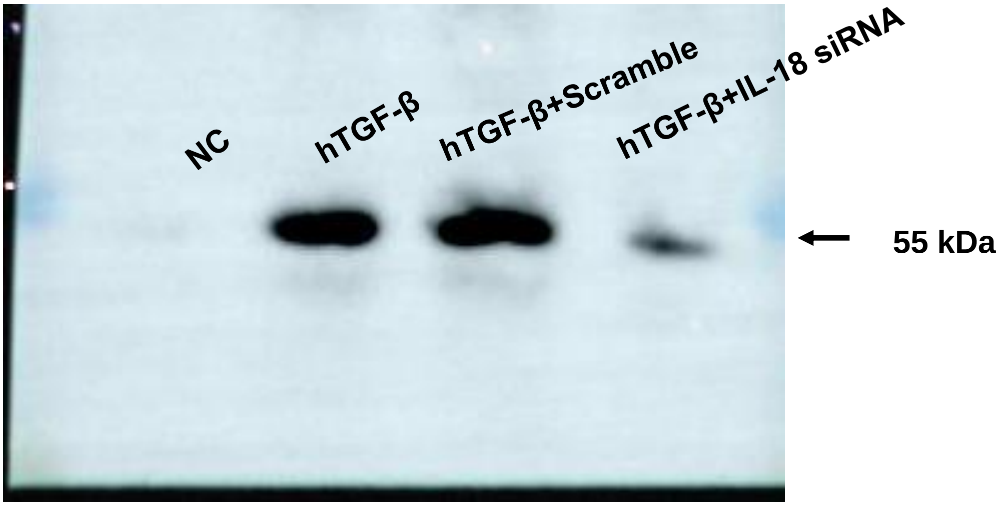

Figure 7 RIPK1

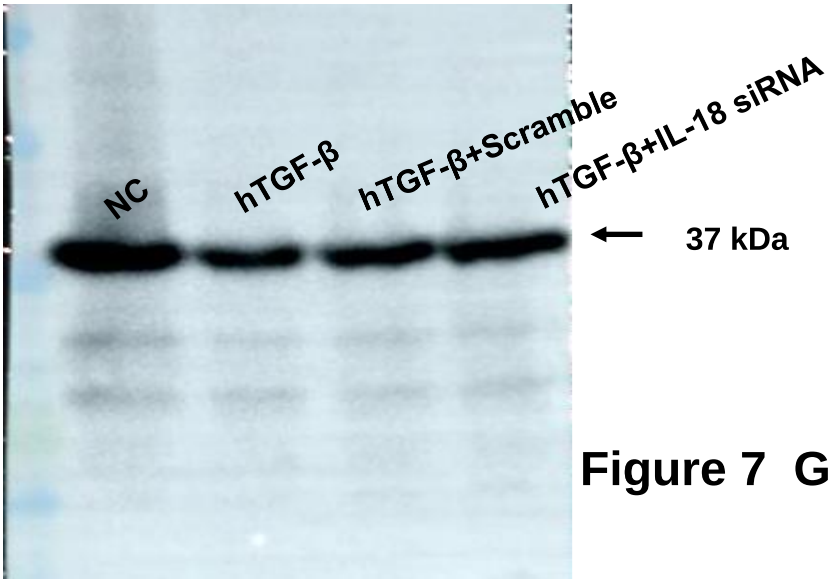

Figure 7 GAPDH

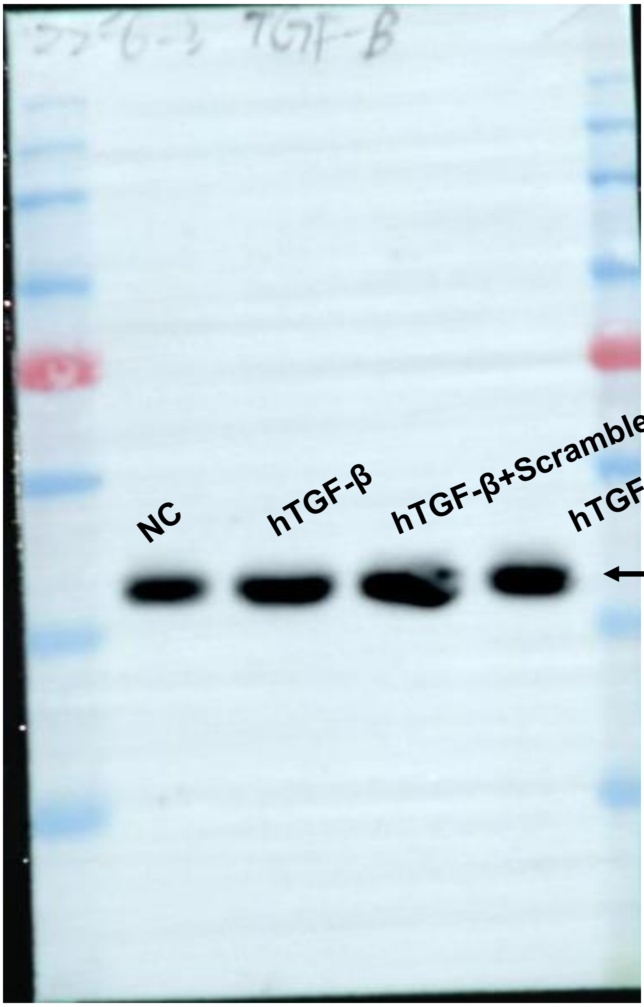

Figure 7 TGF-β1

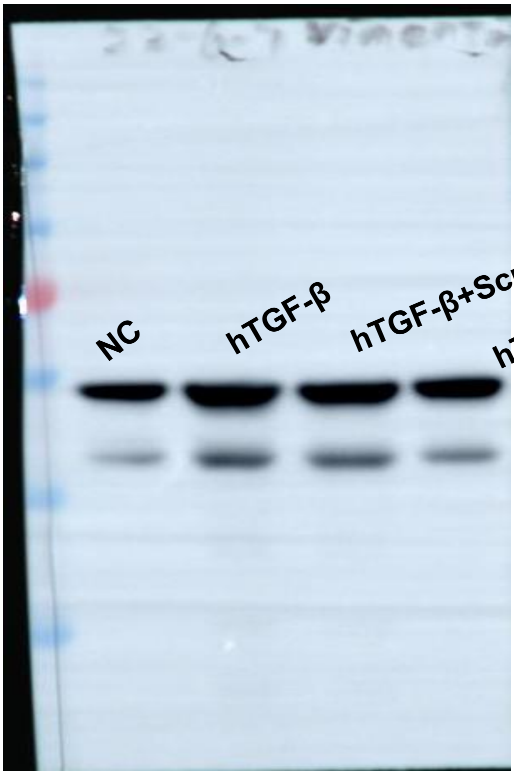

Figure 7 Vimentin

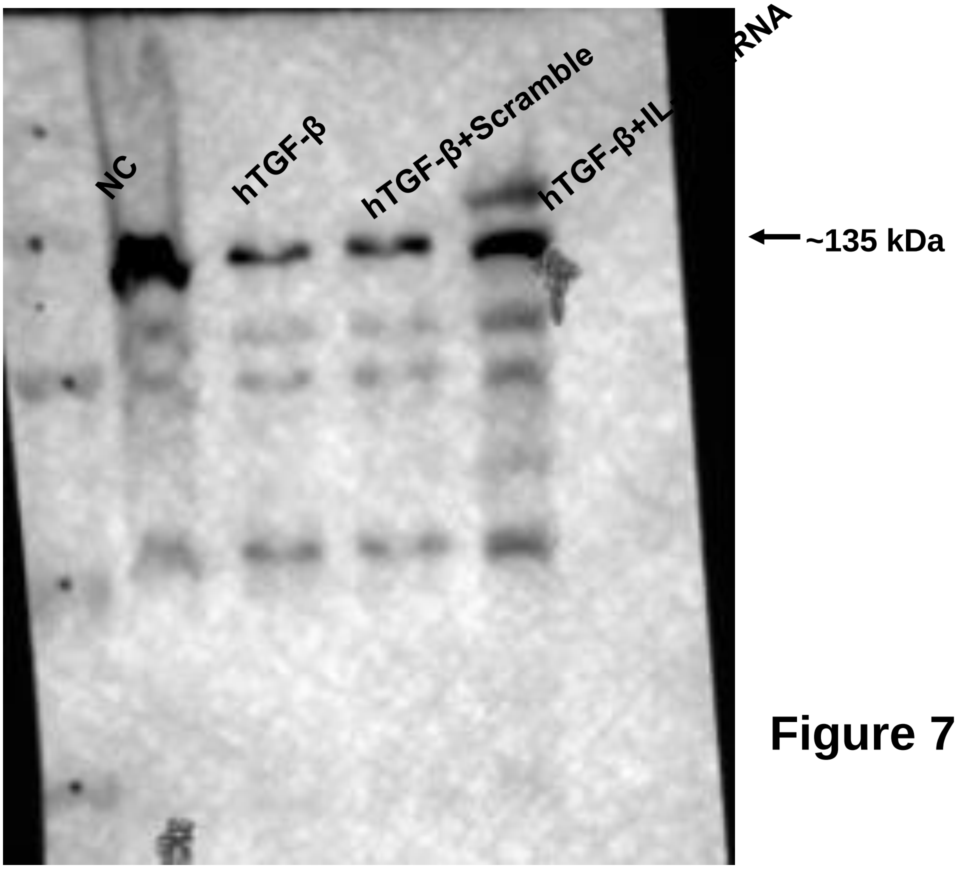

Figure 7 E-cadherin

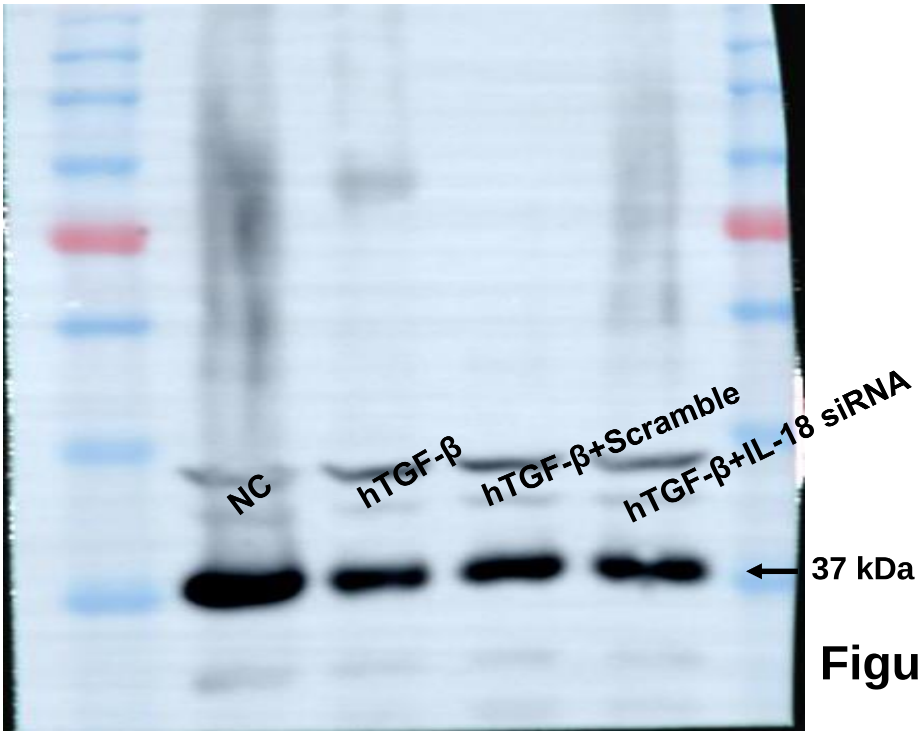

Figure 7 GAPDH

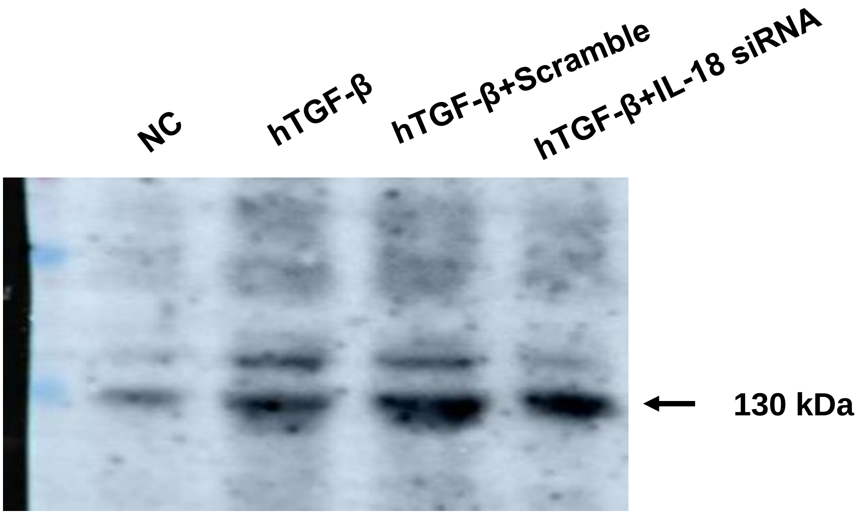

Figure 7 COL-1

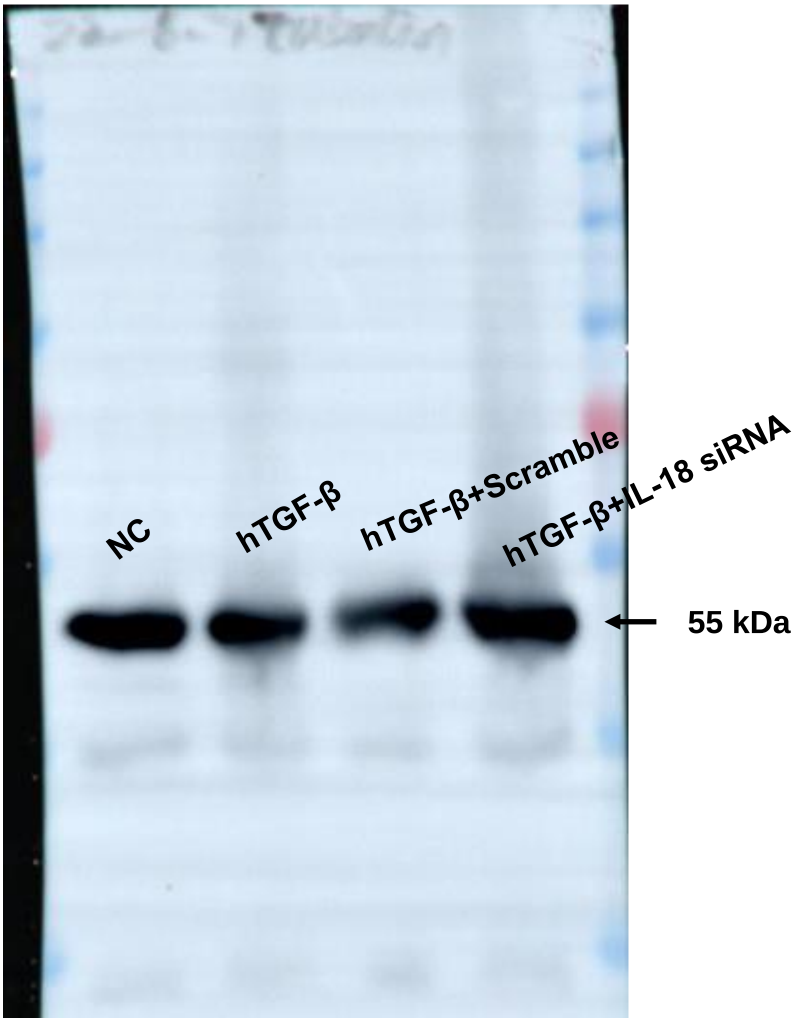

Figure 7 α-TUBULIN
